# Predicting Protein Ligand Binding Sites by Combining Evolutionary Sequence Conservation and 3D Structure

**DOI:** 10.1371/journal.pcbi.1000585

**Published:** 2009-12-04

**Authors:** John A. Capra, Roman A. Laskowski, Janet M. Thornton, Mona Singh, Thomas A. Funkhouser

**Affiliations:** 1 Department of Computer Science, Princeton University, Princeton, New Jersey, United States of America; 2 Lewis-Sigler Institute for Integrative Genomics, Princeton University, Princeton, New Jersey, United States of America; 3 European Bioinformatics Institute, Wellcome Trust Genome Campus, Hinxton, Cambridge, United Kingdom; Max-Planck-Institut für Informatik, Germany

## Abstract

Identifying a protein's functional sites is an important step towards characterizing its molecular function. Numerous structure- and sequence-based methods have been developed for this problem. Here we introduce *ConCavity*, a small molecule binding site prediction algorithm that integrates evolutionary sequence conservation estimates with structure-based methods for identifying protein surface cavities. In large-scale testing on a diverse set of single- and multi-chain protein structures, we show that *ConCavity* substantially outperforms existing methods for identifying both 3D ligand binding pockets and individual ligand binding residues. As part of our testing, we perform one of the first direct comparisons of conservation-based and structure-based methods. We find that the two approaches provide largely complementary information, which can be combined to improve upon either approach alone. We also demonstrate that *ConCavity* has state-of-the-art performance in predicting catalytic sites and drug binding pockets. Overall, the algorithms and analysis presented here significantly improve our ability to identify ligand binding sites and further advance our understanding of the relationship between evolutionary sequence conservation and structural and functional attributes of proteins. Data, source code, and prediction visualizations are available on the *ConCavity* web site (http://compbio.cs.princeton.edu/concavity/).

## Author Summary

Protein molecules are ubiquitous in the cell; they perform thousands of functions crucial for life. Proteins accomplish nearly all of these functions by interacting with other molecules. These interactions are mediated by specific amino acid positions in the proteins. Knowledge of these “functional sites” is crucial for understanding the molecular mechanisms by which proteins carry out their functions; however, functional sites have not been identified in the vast majority of proteins. Here, we present *ConCavity*, a computational method that predicts small molecule binding sites in proteins by combining analysis of evolutionary sequence conservation and protein 3D structure. *ConCavity* provides significant improvement over previous approaches, especially on large, multi-chain proteins. In contrast to earlier methods which only predict entire binding sites, *ConCavity* makes specific predictions of positions in space that are likely to overlap ligand atoms and of residues that are likely to contact bound ligands. These predictions can be used to aid computational function prediction, to guide experimental protein analysis, and to focus computationally intensive techniques used in drug discovery.

## Introduction

Proteins' functions are determined to a large degree by their interactions with other molecules. Identifying which residues participate in these interactions is an important component of functionally characterizing a protein. Many computational approaches based on analysis of protein sequences or structures have been developed to predict a variety of protein functional sites, including ligand binding sites [1]–[3], DNA-binding sites [4], catalytic sites [2],[5], protein-protein interaction interfaces (PPIs) [6],[7] and specificity determining positions [8]–[12]. In this paper, we focus on the task of predicting small molecule binding sites from protein sequences and structures. In addition to aiding in the functional characterization of proteins, knowledge of these binding sites can guide the design of inhibitors and antagonists and provide a scaffold for targeted mutations. Over the past 15 years, a large number of methods for predicting small molecule binding sites have been developed. Structural approaches have used geometric and energetic criteria to find concave regions on the protein surface that likely bind ligands [1], [13]–[21]. Sequence-based approaches, on the other hand, have largely exploited sequence conservation, or the tendency of functionally or structurally important sites to accept fewer mutations relative to the rest of the protein [22].

We introduce *ConCavity*, a new approach for predicting 3D ligand binding pockets and individual ligand binding residues. The *ConCavity* algorithm directly integrates evolutionary sequence conservation estimates with structure-based surface pocket prediction in a modular three step pipeline. In the first step, we score a grid of points surrounding the protein surface by combining the output of a structure-based pocket finding algorithm (e.g., *Ligsite* [16], *Surfnet* [14], or *PocketFinder* [23]) with the sequence conservation values of nearby residues. In the second step, we extract coherent pockets from the grid using 3D shape analysis algorithms to ensure that the predicted pockets have biologically reasonable shapes and volumes. In the final step, we map from the predicted pockets to nearby residues by assigning high scores to residues near high scoring pocket grid points. Using this pipeline, *ConCavity* is able to make predictions of both regions in space that are likely to contain ligand atoms as well as protein residues likely to contact bound ligands.

We demonstrate *ConCavity*'s excellent performance via extensive testing and analysis. First, we show that *ConCavity*, by integrating conservation and structure, provides significant improvement in identifying ligand binding pockets and residues over approaches that use either conservation alone or structure alone; this testing is performed on the diverse, non-redundant LigASite database of biologically relevant binding sites [24]. We find that *ConCavity*'s top predicted residue is in contact with a ligand nearly 80% of the time, while the top prediction of the tested structure-alone and conservation-alone methods is correct in 67% and 57% of proteins respectively. The notable improvement of *ConCavity* over the conservation-alone approach demonstrates that there is significant added benefit to considering structural information when it is available. Second, we demonstrate that *ConCavity* significantly outperforms current publicly available methods [1],[19],[25] that identify ligand binding sites based on pocket finding. Third, we show that *ConCavity* performs similarly when using a variety of pocket detection algorithms [14],[16],[23] or sequence conservation measures [2],[26]. Fourth, we characterize *ConCavity* in a range of situations, and compare its performance in identifying ligand binding sites from apo vs. holo structures as well as in enzymes vs. non-enzymes. Fifth, we test how well *ConCavity* can identify catalytic sites and drug binding sites. Sixth, we examine problematic cases for our approaches, and highlight the difficulty that multi-chain proteins pose for structure-based methods for identifying ligand binding sites. Finally, we demonstrate that our methodological improvements in pocket extraction and residue mapping give our implementations of existing methods a significant gain in performance over the previous versions. In fact, without these improvements, the previous structural approaches do not outperform a simple sequence conservation approach when identifying ligand binding residues. Overall, *ConCavity* significantly advances the state-of-the-art in uncovering ligand binding sites. Our detailed analysis reveals much about the relationship between sequence conservation, structure, and function, and shows that sequence conservation and structure-based attributes provide complementary information about functional importance.

### Further related work

Sequence-based functional site prediction has been dominated by the search for residue positions that show evidence of evolutionary constraint. Amino acid conservation in the columns of a multiple sequence alignment of homologs is the most common source of such estimates (see [22] for a review). Recent approaches that compare alignment column amino acid distributions to a background amino acid distribution outperform many existing conservation measures [2],[27]. However, the success of conservation-based prediction varies based on the type of functional residue sought; sequence conservation has been shown to be strongly correlated with ligand binding and catalytic sites, but less so with residues in protein-protein interfaces (PPIs) [2]. A variety of techniques have been used to incorporate phylogenetic information into sequence-based functional site prediction, e.g., traversing phylogenetic trees [28],[29], statistical rate inference [26], analysis of functional subfamilies [9],[12], and phylogenetic motifs [30]. Recently, evolutionary conservation has been combined with other properties predicted from sequence, e.g., secondary structure and relative solvent accessibility, to identify functional sites [31].

Structure-based methods for functional site prediction seek to identify protein surface regions favorable for interactions. Ligand binding pockets and residues have been a major focus of these methods [1], [13]–[21]. *Ligsite* [16] and *Surfnet* [14] identify pockets by seeking points near the protein surface that are surrounded in most directions by the protein. *CASTp* [17],[19] applies alpha shape theory from computational geometry to detect and measure cavities. In contrast to these geometric approaches, other methods use models of energetics to identify potential binding sites [23], [25], [32]–[34]. Recent algorithms have focused on van der Waals energetics to create grid potential maps around the surface of the protein. *PocketFinder* [23] uses an aliphatic carbon as the probe, and *Q-SiteFinder* [25] uses a methyl group. Our work builds upon geometry and energetics based approaches to ligand binding pocket prediction, but it should be noted that there are other structure-based approaches that do no fit in these categories (e.g., Theoretical Microscopic Titration Curves (THEMATICS) [35], binding site similarity [36], phage display libraries [37], and residue interaction graphs [38]). In contrast to sequence-based predictions, structure-based methods often can make predictions both at the level of residues and regions in space that are likely to contain ligands.

Several previous binding site prediction algorithms have considered both sequence and structure. ConSurf [39] provides a visualization of sequence conservation values on the surface of a protein structure, and the recent *PatchFinder* [40] method automates the prediction of functional surface patches from ConSurf. Spatially clustered residues with high Evolutionary Trace values were found to overlap with functional sites [41], and Panchenko et al. [42] found that averaging sequence conservation across spatially clustered positions provides improvement in functional site identification in certain settings. Several groups have attempted to identify and separate structural and functional constraints on residues [43],[44]. Wang et al. [45] perform logistic regression on three sequence-based properties and predict functional sites by estimating the effect on structural stability of mutations at each position. Though these approaches make use of protein structures, they do not explicitly consider the surface geometry of the protein in prediction. Geometric, chemical, and evolutionary criteria have been used together to define motifs that represent known binding sites for use in protein function prediction [46]. Machine learning algorithms have been applied to features based on sequence and structure [47],[48] to predict catalytic sites [5], [49]–[51] and recently to predict drug targets [52] and a limited set of ligand and ion binding sites [53]–[55]. Sequence conservation has been found to be a dominant predictor in these contexts.

Most similar to *ConCavity* are two recent approaches to ligand binding site identification that have used evolutionary conservation in a post-processing step to rerank [1] or refine [56] geometry based pocket predictions. In contrast, *ConCavity* integrates conservation directly into the search for pockets. This allows it to identify pockets that are not found when considering structure alone, and enables straightforward analysis of the relationship between sequence conservation, structural patterns, and functional importance.

## Results

### Preliminaries

For simplicity of exposition, we begin by comparing *ConCavity*'s performance to a representative structural method and a representative conservation method. We use *Ligsite^+^* as the representative structure-based method, and refer to it as “*Structure*”. *Ligsite^+^* is our implementation (as indicated by superscript “+”) of a popular geometry based surface pocket identification algorithm. We demonstrate in the Methods section that *Ligsite^+^* provides a fair representation of these methods. We choose *Jensen-Shannon divergence* (*JSD*) to represent conservation methods and refer to it as “*Conservation*.” *JSD* has been previously shown to provide state-of-the-art performance in identifying catalytic sites and ligand binding sites [2]. We have developed three versions of *ConCavity* that integrate evolutionary conservation into different surface pocket prediction algorithms (*Ligsite* [16], *Surfnet* [14], or *PocketFinder* [23]). When the underlying algorithm is relevant, we refer to these versions as *ConCavity^L^*, *ConCavity^S^*, and *ConCavity^P^*. However, for simplicity, we will use *ConCavity^L^* as representative of these approaches and call it “*ConCavity*.”

*ConCavity* and *Structure* produce predictions of ligand binding pockets and residues. The pocket predictions are given as non-zero values on a regular 3D grid that surrounds the protein; the score associated with each grid point represents an estimated likelihood that it overlaps a bound ligand atom. Similarly, each residue in the protein sequence is assigned a score that represents its likelihood of contacting a bound ligand. *Conservation* only makes residue-level predictions, because it does not consider protein structure. All methods are evaluated on 332 proteins from the non-redundant LigASite 7.0 dataset [24]. To evaluate pocket identification performance, we predict ligand locations on the the holo version of the dataset, in order to use the bound ligands' locations as positives. When evaluating residue predictions, we predict ligand binding residues on the apo structures, and the residues annotated as ligand binding (as derived from the holo structures) are used as positives.

We quantify the overall performance of each method's predictions in two ways. First, for both pocket and residue prediction, we generate precision-recall (PR) curves that reflect the ability of each method's grid and residue scores to identify ligand atoms and ligand binding residues, respectively. (Just as residues are assigned a range of ligand binding scores, grid points in predicted pockets get a range of scores, since there may be more evidence that a ligand is bound in one part of a pocket than another.) Second, for each set of predicted pockets (corresponding to groups of non-zero values in the 3D grid), we consider how well they overlap known ligands via the Jaccard coefficient. The Jaccard coefficient captures the tradeoff between precision and recall by taking the ratio of the intersection of the predicted pocket and the actual ligand over their union. The Jaccard coefficient ranges between zero and one, and a high value implies that the prediction covers the ligand well and has a similar volume. We assess the significance of the difference in performance of methods on the dataset with respect to a given statistic via the Wilcoxon rank-sum test.

### Integrating evolutionary sequence conservation and structure-based pocket finding to predict ligand-binding sites improves on either approach alone

**Figure 1:**
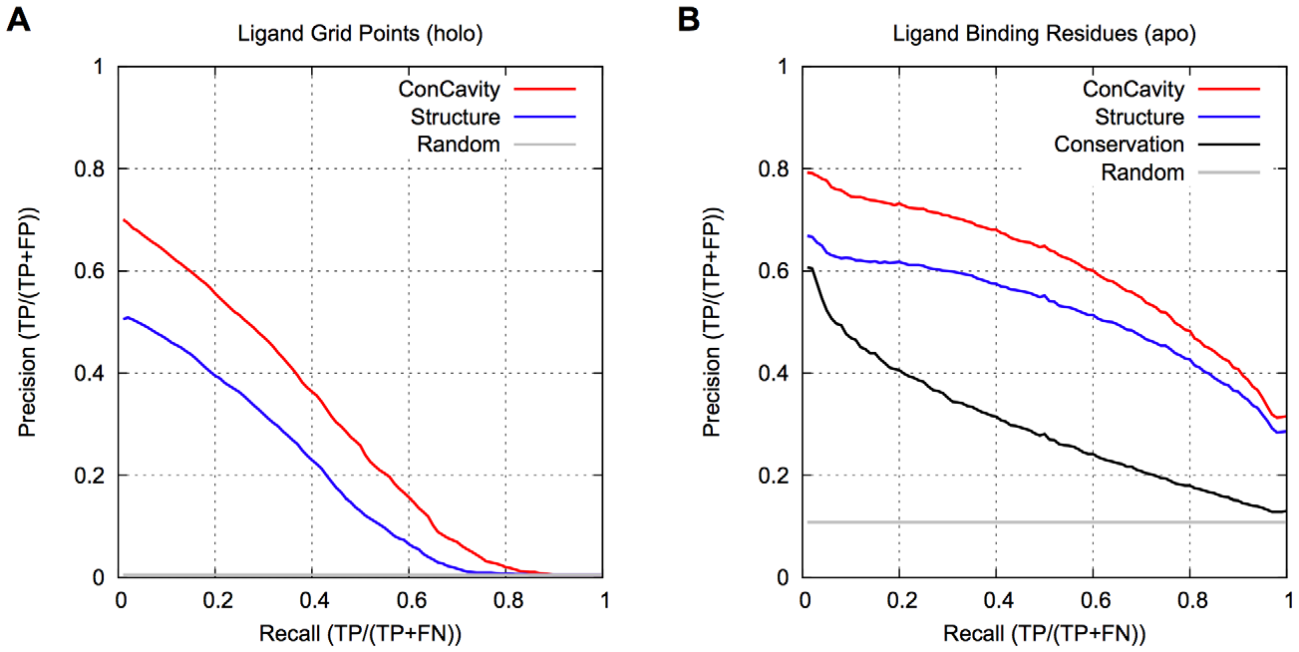
Ligand binding site prediction performance. (A) PR curves for prediction of the spatial location of biologically relevant bound ligands. (B) PR curves for ligand binding residue prediction. Our *ConCavity* algorithm, which combines sequence conservation with structure-based predictors, significantly outperforms either of the constituent methods at both tasks. Prediction based on structural information alone outperforms considering sequence conservation alone. Comparing (A) and (B), we see that accurately predicting the location of all ligand atoms is harder for the methods than finding all the contacting residues. *Random* gives the expected performance of a method that randomly ranks grid points and residues. *Conservation* could not be included in (A), because it only predicts at the residue level. The curves are based on binding sites in 332 proteins from the non-redundant LigASite 7.0 dataset.

Figure 1 compares *ConCavity* with its constituent structure and conservation based components. Figure 1A shows that, within predicted pockets, grid points with higher scores are more likely to overlap the ligand, and that the significant improvement of *ConCavity* over *Structure* (p<2.2e−16) exists across the range of score thresholds. Figure 1B demonstrates that the superior performance of *ConCavity* holds when predicting ligand binding residues as well (p = 6.80e−13). *ConCavity*'s ability to identify ligand binding residues is striking: across this diverse dataset, the first residue prediction of *ConCavity* will be in contact with a ligand in nearly 80% of proteins. *ConCavity* also maintains high precision across the full recall range: precision of 65% at 50% recall and better than 30% when all ligand-binding residues have been identified. As mentioned above, this large improvement exists when predicting ligand locations as well; however, the PR curves illustrate that fully identifying a ligand's position is more difficult for each of the methods than finding all contacting residues.

The ligand overlap statistics presented in Table 1 also demonstrate the superior performance of *ConCavity*. In nearly 95% of structures, *ConCavity*'s predictions overlap with a bound ligand. *Structure*'s predictions overlap ligands in nearly 92% of the proteins considered. The differences between the methods become more stark when we examine the magnitude of these overlaps. Both *ConCavity* and *Structure* predict pockets with total volume (Prediction Vol.) similar to that of all relevant ligands (Ligand Vol.), but *ConCavity*'s pockets overlap a larger fraction of the ligand volume. Thus *ConCavity* has a significantly higher Jaccard coefficient (p<2.2e−16). This suggests that the integration of sequence conservation with structural pocket identification results in more accurate pockets than when using structural features alone.

**Table 1:** The overlap between predicted pockets and bound ligands in holo protein structures from the LigASite database.

Method	Fraction with Ligand Overlap	Prediction Vol. (Å^3^)	Ligand Vol. (Å^3^)	Prediction 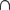 Ligand (Å^3^)	Prediction 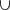 Ligand (Å^3^)	Jaccard coefficient
*Structure*	0.92	1806.8	1977.2	426.9	3357.1	0.197
*ConCavity*	0.95	1806.9	1977.2	647.6	3136.5	0.257

The first column gives the fraction of proteins for which a method's predictions overlap a ligand. The second column (Prediction Vol.) lists the average volume of the predicted pockets for each protein, while the third column (Ligand Vol.) lists the average volume of ligands observed in the structure. The next columns give the average volumes of the Intersection and Union of the predictions and ligands and the Jaccard coefficient (Intersection/Union). *ConCavity* and *Structure* predict pockets of similar sizes---both use a similar pocket volume threshold---but *ConCavity*'s predictions overlap more of the bound ligands. *ConCavity*'s higher Jaccard coefficient demonstrates that it better manages the tradeoff between precision and recall.

Figure 1B also provides a direct comparison of ligand binding site prediction methods based on sequence conservation with those based on structural features. *Structure* outperforms *Conservation*, a state-of-the-art method for estimating sequence conservation. Protein residues can be evolutionarily conserved for a number of reasons, so it is not surprising that *Conservation* identifies many non-ligand-binding residues, and thus, does not perform as well as *Structure*.

### *ConCavity*'s improvement comes from integrating complementary information from evolutionary sequence conservation and structure-based pocket identification

**Figure 2:**
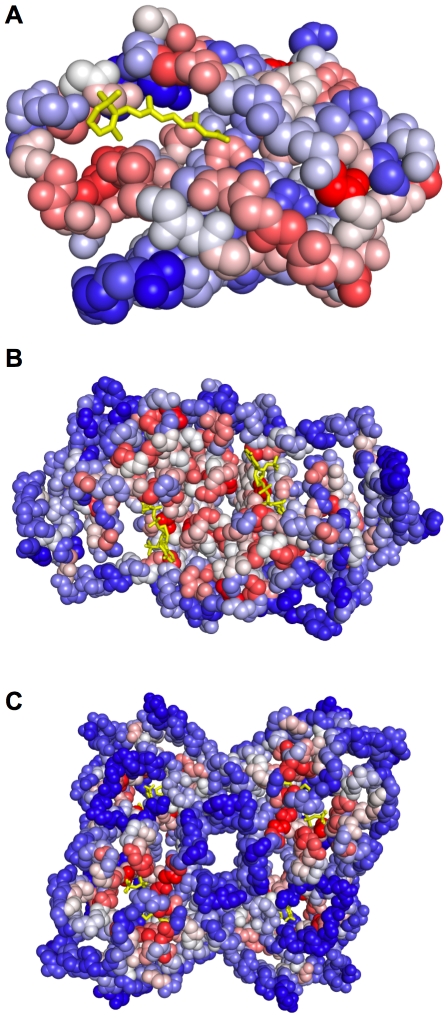
Evolutionary sequence conservation mapped to the surface of three example proteins. (A) Cellular retinoic acid-binding protein II (PDB: 3CWK). (B) Delta1-piperideine-2-carboxylate reductase (PDB: 2CWH). (C) Thiamin phosphate synthase (PDB: 1G6C). Warmer colors indicate greater evolutionary conservation; the most conserved residues are colored dark red, and the least conserved are colored dark blue. Ligands are rendered with yellow sticks, and protein backbone atoms are shown as spheres. In general, *Conservation* gives the highest scores to residues near ligands, but high scoring residues are found throughout each structure. The predictions of *Structure* and *ConCavity* for these proteins are given in Figure 3.

**Figure 3:**
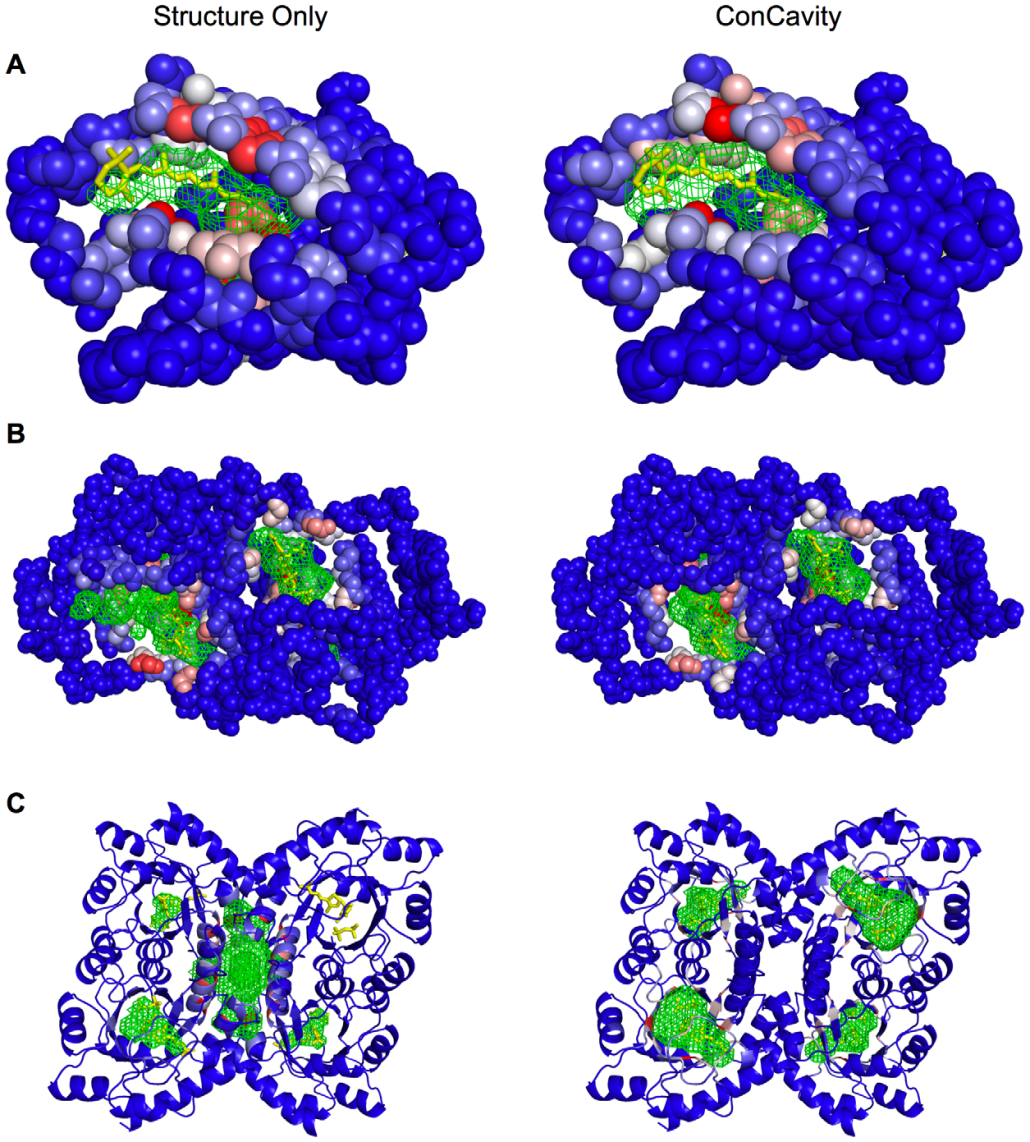
Comparison of the binding site predictions of *Structure* and *ConCavity* on three example proteins. The three proteins presented here correspond to those shown in Figure 2. In each pane, ligand binding residue scores have been mapped to the protein surface. Warmer colors indicate a higher binding score. Pocket predictions are shown as green meshes. (A) PDB: 3CWK. Both methods identify the binding site, but by considering conservation information (Figure 2A), *ConCavity* more accurately traces the ligand. (B) PDB: 2CWH. *Structure* significantly overpredicts the extent of the ligand in the bottom left corner as well as predicting an additional pocket on the reverse of the protein. *ConCavity* predicts only the two ligand binding pockets. (C) PDB: 1G6C. In order to visualize the predictions more clearly, only the secondary structure diagram of the protein is shown. This example illustrates the difficulty presented by multichain proteins; there are many cavities in the structure, but not all bind ligands. *Structure* identifies some of the relevant pockets, but focuses on the large, non-binding central cavity formed between the chains. Referring to this protein's conservation profile (Figure 2C), we see that the ligand binding pockets exhibit high conservation while the non-binding pockets do not. As a result, *ConCavity* selects only the relevant binding pockets. In each example, *ConCavity* selects the binding pocket(s) out of all potential pockets and more accurately traces the ligands' locations in these pockets.

Figures 2 and 3 present pocket and residue predictions of *Conservation*, *Structure*, and *ConCavity* on three example proteins. In general, different types of positions are predicted by *Conservation* and *Structure*. If we consider the number of known ligand binding residues for each protein in the dataset, and take this number of top predictions for the *Structure* and *Conservation* methods, the overlap is only 26%. The residues predicted by sequence conservation are spread throughout the protein (Figure 2); ligand-binding residues are often very conserved, but many other positions are highly conserved as well due to other functional constraints. In contrast, the structure-based predictions are strongly clustered around surface pockets (Figure 3, left column); many of these residues near pockets are not evolutionarily conserved. However, these features provide largely complementary information about importance for ligand binding. Over the entire dataset, 68% of residues predicted by both *Conservation* and *Structure* are in contact with ligands, while only 16% and 43% of those predicted by only conservation or structure respectively are ligand binding. *ConCavity* takes advantage of this complementarity to achieve its dramatic improvement; it gives high scores to positions that show evidence of both being in a well-formed pocket and being evolutionarily conserved.

The examples of Figures 2 and 3 illustrate this and highlight several common patterns in *ConCavity*'s improved predictions. For 3CWK, a cellular retinoic acid-binding protein, *Structure* and *ConCavity*'s residue predictions center on the main ligand binding pocket (Figure 3A), while *Conservation* gives high scores to some positions in the binding site, but also to some unrelated residues (Figure 2A). Looking at the ligand location predictions (green meshes in Figure 3A), *Structure* and *ConCavity* both find the pocket, but the signal from conservation enables *ConCavity* to more accurately trace the ligand's location. This illustrates how the pattern of functional conservation observed at the protein surface influences the shape of the predicted pocket. Ligands often do not completely fill surface pockets; if the contacting residues are conserved, our approach can suggest a more accurate shape.

The results for 2CWH (Figure 3B) and 1G6C (Figure 3C) demonstrate that *ConCavity* can predict dramatically different sets of pockets than are obtained when considering structure alone. In 2CWH, both methods identify the ligands, but *Structure* over-predicts the bottom left binding pocket and predicts an additional pocket that does not have a ligand bound. *ConCavity* traces the ligands more closely and does not predict any additional pockets. *Structure* performs quite poorly on the tetramer 1G6C: it predicts several pockets that do not bind ligands; it fails to completely identify several ligands; and it misses one ligand entirely. In stark contrast, *ConCavity*'s four predicted pockets each accurately trace a ligand. The incorporation of conservation resulted in the accurate prediction of a pocket in a region where no pocket was predicted using structure alone. Images of predictions for all methods on all proteins in the dataset are available in the Text S1 file, and *ConCavity*'s predictions for all structures in the Protein Quaternary Structure (PQS) database are available online.

### *ConCavity* significantly outperforms available prediction servers

We now compare the performance of *ConCavity* to several existing ligand binding site identification methods with publicly available web servers. *LigsiteCS* [1] is an updated version of geometry-based *Ligsite*, and *LigsiteCSC* [1] is a similar structural method that considers evolutionary conservation information. *Q-SiteFinder* [25] estimates van der Waals interactions between the protein and a probe in a fashion similar to *PocketFinder. CASTp* [19] is a geometry-based algorithm for finding pockets based on analysis of the protein's alpha shape. Each of the servers produces a list of predicted pockets represented by sets of residues; however, none of them provide a full 3D representation of a predicted pocket. As a result, we assess their ability to predict ligand binding residues. See the Methods section for more information on the generation and processing of the servers' predictions. In brief, the residues predicted by each server are ranked according to the highest ranking pocket to which they are assigned, i.e., all residues from the first predicted pocket are given a higher score than those from the second and so on. We re-implemented the conservation component of *LigsiteCSC*, because the conservation-based re-ranking option on the web server did not work for many of the proteins in our dataset. We used *JSD* as the conservation scoring method.

**Figure 4:**
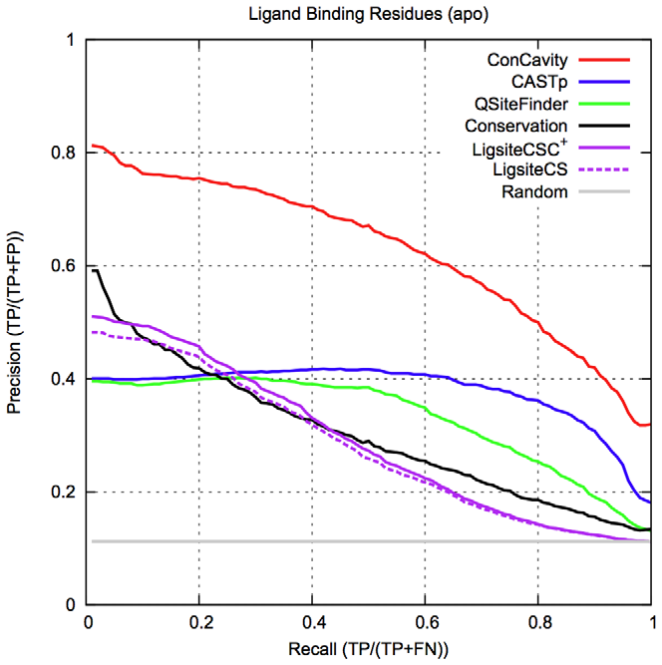
Comparison of *ConCavity* with publicly available ligand binding site prediction servers. *ConCavity* significantly outperforms each previous method at the prediction of ligand binding residues. The existing servers focus on the task of pocket prediction, and return sets of residues that represent binding pocket predictions. They do not give different scores to these individual residues. In contrast, *ConCavity* assigns each residue a likelihood of binding, and thus residues in the same predicted pocket can have different scores. This ability and the direct integration of sequence conservation are the major sources of *ConCavity*'s improvement. *Conservation*, the method based solely on sequence conservation, is competitive with these previous structural approaches. This figure is based on 234 proteins from the LigASite apo dataset for which we were able to obtain predictions from all methods.

Figure 4 presents the ligand binding residue PR-curves for each of these methods. *ConCavity* significantly outperforms *LigsiteCS*, *LigsiteCSC^+^*, *Q-SiteFinder*, and *CASTp* (p<2.2e−16 for each). Surprisingly, *Conservation* is competitive with these structure-based approaches. Several of the servers did not produce predictions for a small subset of the proteins in the database, e.g., the *Q-SiteFinder* server does not accept proteins with more than 10,000 atoms. Figure 4 is based on 234 proteins from the LigASite dataset for which were able to obtain and evaluate predictions for all methods. Thus the curve for *ConCavity* is slightly different than those found in the other figures, but its performance does not change significantly.

*LigsiteCSC^+^* is the previous method most similar to *ConCavity*; it uses sequence conservation to rerank the pockets predicted by *LigsiteCS*. *LigsiteCSC^+^* provides slight improvement over *LigsiteCS*, but the improvement is dwarfed by that of *ConCavity* over *Structure* (Figure 1). This illustrates the benefit of incorporating conservation information directly into the search for pockets in contrast to using conservation information to post-process predicted pockets.

The poor performance of these previous methods at identifying ligand binding residues is due in part to the fact that they do not distinguish among the residues near a predicted binding pocket. The entire pocket is a useful starting place for analysis, but many residues in a binding pocket will not actually contact the ligand. Knowledge of the specific ligand binding residues is of most interest to researchers. The predictions of our methods reflect this---residues within the same pocket can receive different ligand binding scores. The inability of previous methods to differentiate residues in a pocket from one another is one reason why we elect to use our own implementations of previous structure-based methods as representatives of these approaches in all other comparisons. See the Methods section for more details.

We tested an additional approach for combining sequence conservation with structural information that was suggested by the observation that clusters of conserved residues in 3D often overlap with binding sites [41],[42]. Briefly, the method performs a 3D Gaussian blur of the conservation scores of each residue, and assigns each residue the maximum overlapping value. Thus residues nearby in space to other conserved residues get high scores. This approach improved on considering conservation alone, but was not competitive with *ConCavity* (Text S1). We also considered the clusters of conserved residues generated by the *Evolutionary Trace (ET) Viewer* [57]. The clusters defined at 25% protein coverage were ranked by size, and residues within the clusters were ranked by their raw *ET* score. This approach did not perform as well as the above clustering algorithm (data not shown), and was limited to single chain proteins, because *ET* returns predictions for only one chain of multi-chain proteins.

### *ConCavity* performs similarly for geometry and energetics based grid creation methods

In the previous sections, we used *ConCavity^L^*, which integrates evolutionary sequence conservation estimates from the Jensen-Shannon divergence (JSD) into *Ligsite^+^*, to represent the performance of the *ConCavity* approach. However, our strategy for combining sequence conservation with structural predictions is general; it can be used with a variety of grid-based surface pocket identification algorithms and conservation estimation methods.

**Figure 5:**
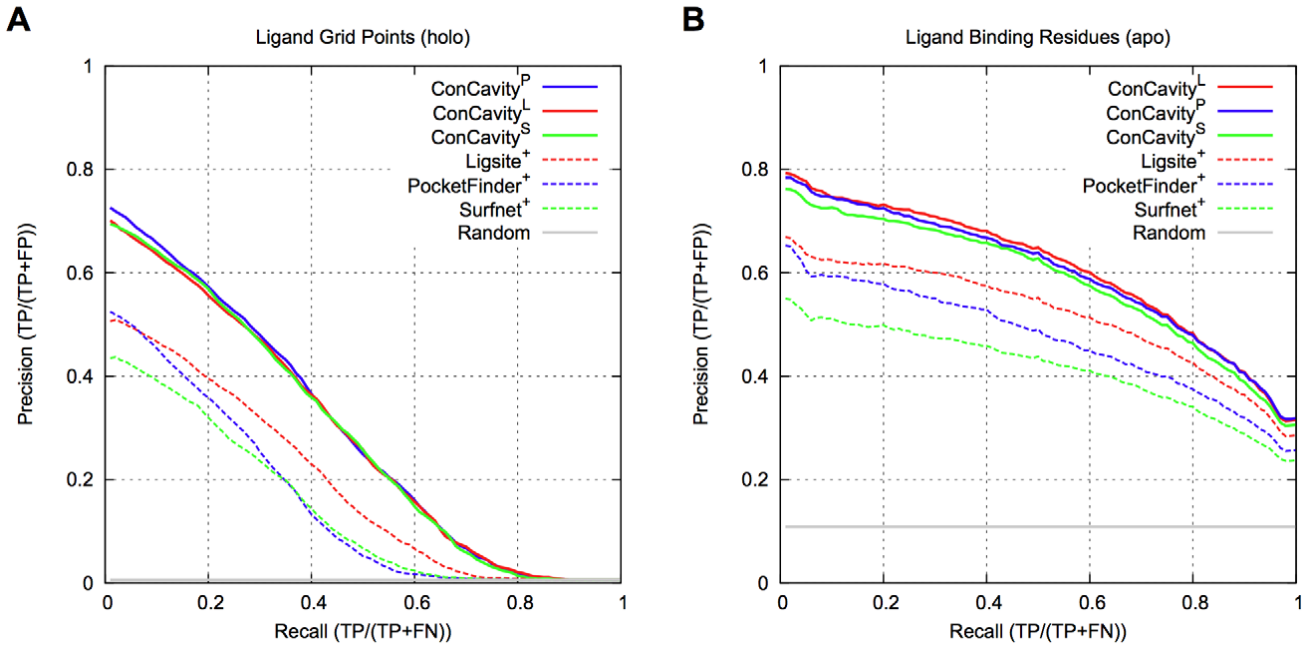
Comparison of different versions of *ConCavity.* *ConCavity* provides a general framework for binding site prediction. We use *Ligsite^+^* -based *ConCavity* as representative, but it is possible to use other algorithms in *ConCavity*. This figure compares the PR curves for three versions (*ConCavity^L^*, *ConCavity^P^*, *ConCavity^S^* )---each based on integrating sequence conservation with a different grid creation strategy (*Ligsite^+^*, *PocketFinder^+^*, or *Surfnet^+^)*. All three versions perform similarly, and all significantly outperform the methods based on structure analysis alone (dashed lines). These conclusions hold for both ligand binding pocket (A) and ligand binding residue (B) prediction.

Figure 5 gives PR-curves that demonstrate that *ConCavity* provides excellent performance whether the structural approaches are based on geometric properties (*Ligsite^+^*, *Surfnet^+^*) or energetics (*PocketFinder^+^*). The significant improvement holds for predicting both ligand locations in space (p<2.2e−16 for each pair) (Figure 5A) and ligand binding residues (p = 6.802e−13 for *Ligsite^+^*, p<2.2e−16 for *PocketFinder^+^*, p<2.2e−16 for *Surfnet^+^*) (Figure 5B). The three *ConCavity* versions perform similarly despite the variation in performance between *Ligiste^+^*, *Surfnet^+^*, and *Pocketfinder^+^*. In the following sections we will include performance statistics for all three methods when space and clarity allow. When not presented here, results for all methods are available in the supplementary file Text S1.

We have also found that *ConCavity* achieves similar performance when a different state-of-the-art method [26] is used to score evolutionary sequence conservation (Text S1).

### Structure-based methods have difficulty with multi-chain proteins

**Figure 6:**
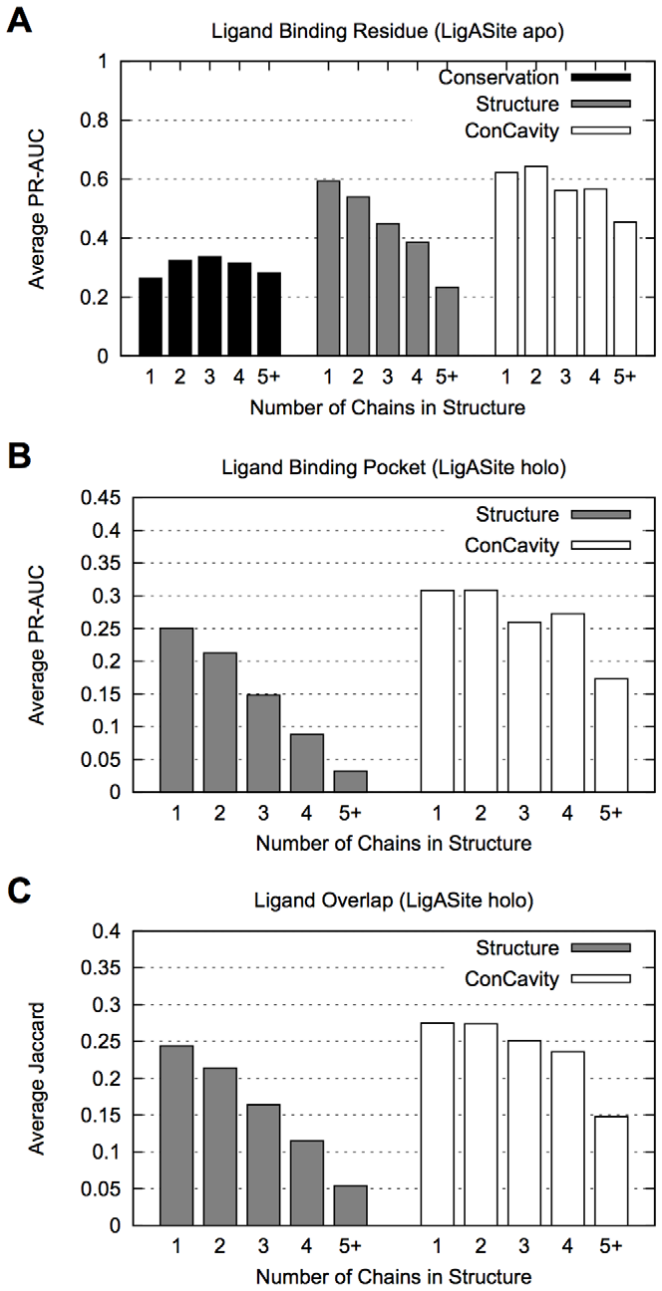
Ligand-binding site identification performance by number of chains in structure. (A) The average area under the precision-recall curve (PR-AUC) for predicting ligand binding residues on each set of structures. (B) The average PR-AUC for ligand binding pocket identification. (C) The average Jaccard coefficient of the overlap of the predicted pockets with bound ligands. Methods based on structure alone have an increasingly difficult time distinguishing among ligand-binding pockets and non-ligand-binding gaps between chains as the number of chains in the protein increases. This trend is clear in each evaluation. *Conservation*'s performance does not exhibit this effect (A). In fact, *Conservation* outperforms *Structure* on proteins with five or more chains. The integration of sequence conservation and pocket prediction in *ConCavity* improves performance in each chain based partition in each evaluation, and *ConCavity* sees only a modest decrease in performance on proteins with multiple chains. *Conservation* alone could not be included in (B) and (C), because it does not make pocket predictions. Note that the y-axes in the figures do not all have the same scale. The number of structures per chain group: 1 chain: 143, 2 chains: 112, 3 chains: 18, 4 chains: 35, 5 or more chains: 24.

Proteins consisting of multiple subunits generally have more pockets than single-chain proteins due to the gaps that often form between chains. To investigate the effect of structural complexity on performance, we partitioned the dataset according to the number of chains present in the structure predicted by the Protein Quaternary Structure (PQS) server [58] and performed our previous evaluations on the partitioned sets. Figure 6 gives these statistics for *ConCavity*, *Structure*, and *Conservation*. To enable side-by-side comparison, we report the area under the PR curves (PR-AUC) rather than giving the full curves.

As the number of chains in the structure increases, there is a substantial decrease in the performance of *Structure*. The pattern is seen both when predicting ligand binding residues (Figure 6A) and pockets (Figure 6B, C). This effect is so large that, for proteins with five or more chains, *Conservation* outperforms *Structure*. The number of chains in the protein has little effect on *Conservation*'s performance. The performance of *Random* on proteins with a small number of chains is slightly worse than on proteins with many chains (e.g., Residue PR-AUC for 1 chain: 0.097, 2 chains: 0.110, 3 chains: 0.127, 4 chains: 0.119, 5+ chains: 0.142), so the drop in *Structure*'s performance is not the result of the proportion of positives in each set. These observations emphasize the importance of including multi-chain proteins in the evaluation.

The homo-tetramer 1G6C in Figure 3C provides an illustrative example of the failure of *Structure* on multi-chain proteins. There is a large gap between the chains in the center of the structure, and several additional pockets are formed at the interface of pairs of contacting chains. As seen in the figure, the large central cavity does not bind a ligand; however, it is the largest pocket predicted by *Structure*. This is frequently observed among the predictions. While some pockets between protein chains are involved in ligand binding, many of them are not. As the number of chains increases, so does the number of such potentially misleading pockets.

By incorporating sequence conservation information, *ConCavity* accurately identifies ligand binding pockets in multi-chain proteins. The conservation profile on the surface of 1G6C provides a clear example of this; the pockets that exhibit sequence conservation are those that bind ligands (Figure 2C). 1G6C is not an exception. *ConCavity* provides significant performance improvement for each partition of the dataset in all three evaluations, and greatly reduces the effect of the large number of non-ligand-binding pockets in multi-chain proteins on performance. *ConCavity* also provides improvement over *Structure* on the set of one chain proteins. This is notable because these proteins do not have between-chain gaps, so the improvement comes from tracing ligands and selecting among intra-chain pockets more accurately than using structural information alone (as in Figure 3A).

### *ConCavity* performs well on both apo and holo structures

The binding of a ligand induces conformational changes to a protein [59]. As a result, the 3D structure of the binding site can differ between structures of the same protein with a ligand bound (holo) and not bound (apo). In the holo structures, the relevant side-chains are in conformations that contact the ligand, and this often defines the binding pocket more clearly than in apo structures. To investigate the effect of the additional information provided in holo structures on performance, we evaluated the methods on both sets (Table 2).

**Table 2:** Area under the Precision-Recall curve (PR-AUC) for ligand-binding residue prediction methods on apo (unbound) and holo (bound) versions of LigASite.

	Residue PR-AUC	
Method	apo	holo
*ConCavity^L^*	0.608	0.657
*ConCavity^P^*	0.601	0.646
*ConCavity^S^*	0.586	0.648
*Ligsite^+^*	0.519	0.552
*PocketFinder^+^*	0.472	0.514
*Surfnet^+^*	0.416	0.481
*Random*	0.109	0.095

All methods perform better on holo structures than apo structures, but the drop in performance is not dramatic, and the relative ranking of the methods is the same across both datasets.

As expected, all methods performed better on the holo (bound) structures than the corresponding apo (unbound) structures. However, all previous conclusions hold whether considering apo structures or holo structures; the ranking of the methods is consistent, and the improvement provided by considering conservation is similarly large. PR curves for this comparison are given in the supplementary file Text S1. We will continue to report residue prediction results computed using the apo structures when possible in order to accurately assess the performance of the algorithms in the situation faced by ligand binding site prediction methods in the real world.

### The methods better identify ligand binding sites in enzymes than non-enzymes

The LigASite apo dataset contains protein molecules that carry out a range of different functions. Enzymes are by far the most common; they make up 254 of the 332 proteins in the dataset. The remaining 78 non-enzyme ligand binding proteins are involved in a wide variety of functions, e.g., transport, signaling, nucleic acid binding, and immune system response.

Table 3 compares the performance of the ligand binding site prediction methods on enzymes and non-enzymes. There is more variation within each method's performance on non-enzyme proteins, and all methods perform significantly better on the enzymes (e.g., p = 3.336e−4 for *ConCavity^L^* ). Active sites in enzymes are usually found in large clefts on the protein surface and consistently exhibit evolutionary sequence conservation [60],[61], so even though enzymes bind a wide array of substrates, these common features may simplify prediction when compared to the variety of binding mechanisms found in other proteins.

**Table 3:** Ligand binding residue identification in enzymes and non-enzymes (LigASite apo).

	Residue PR-AUC	
Method	Enzyme	Non-enzyme
*ConCavity^L^*	0.647	0.480
*ConCavity^P^*	0.642	0.466
*ConCavity^S^*	0.624	0.461
*Ligsite^+^*	0.541	0.451
*PocketFinder^+^*	0.494	0.399
*Surfnet^+^*	0.430	0.370
*Conservation*	0.318	0.216
*Random*	0.104	0.123

All methods are better at identifying binding residues in enzymes than in non-enzymes. The *ConCavity* methods achieve the best performance on both datasets, but incorporating conservation information provides less improvement in non-enzymes.

Despite the drop in performance on non-enzyme proteins, the main conclusions from the earlier sections still hold. However, the improvement provided by *ConCavity* is not as great on the non-enzymes. This could be the result of the more complex patterns of conservation found in non-enzyme proteins, and the comparatively poor performance of *Conservation* in this setting. It is also possible that *Ligsite^+^*'s approach is particularly well suited to identifying binding sites in non-enzymes. Overall, these results highlight the importance of using a diverse dataset to evaluate functional site predictions.

### *ConCavity* improves identification of drug binding sites

Knowledge of small molecule binding sites is of considerable use in drug discovery and design. Many of the techniques used to screen potential targets, e.g., docking and virtual screening, are computationally intensive and feasible only when focused on a specific region of the protein surface. Structure based surface cavity identification algorithms can guide analysis in such situations [52].

To test *ConCavity*'s ability to identify drug binding sites, we evaluated it on a set of 98 protein-drug complexes [62]. The superior performance provided by *ConCavity* over *Structure* on the diverse set of proteins considered above suggests that *ConCavity* would likely be useful in the drug screening pipeline. Table 4 compares the ligand overlap PR-AUC and Jaccard coefficient for the three versions of *ConCavity* and their structure-based analogs. Each *ConCavity* method significantly improves on the methods that only consider structural features (e.g., p = 1.25e−6 on overlap PR-AUC and p = 2.06e−6 on Jaccard for *ConCavity^L^*). While the improvement is not quite as large on this dataset as that seen on the more diverse LigASite dataset, it is still significant. It is possible that this is due to the fact that drug compounds are not the proteins' natural ligands; the evolutionary conservation of the residues in binding pockets may reflect the pressures related to binding the actual ligands rather than the drugs.

**Table 4:** Drug binding site identification.

Method	Grid Value PR-AUC	Jaccard coefficient
*ConCavity^L^*	0.271	0.240
*ConCavity^P^*	0.263	0.222
*ConCavity^S^*	0.278	0.236
*Ligsite^+^*	0.217	0.207
*PocketFinder^+^*	0.195	0.191
*Surfnet^+^*	0.170	0.183
*Random*	0.006	N/A

This table compares the average grid value precision-recall AUC and the average Jaccard coefficient of prediction-ligand overlap for *ConCavity* and methods based on structural analysis alone on a set of 98 protein-drug complexes. Integrating sequence conservation and structure-based pocket finding improves the identification of drug binding sites. *Conservation* is not included in this evaluation, because it does not make pocket-level predictions.

### Examples of difficult structures

**Figure 7:**
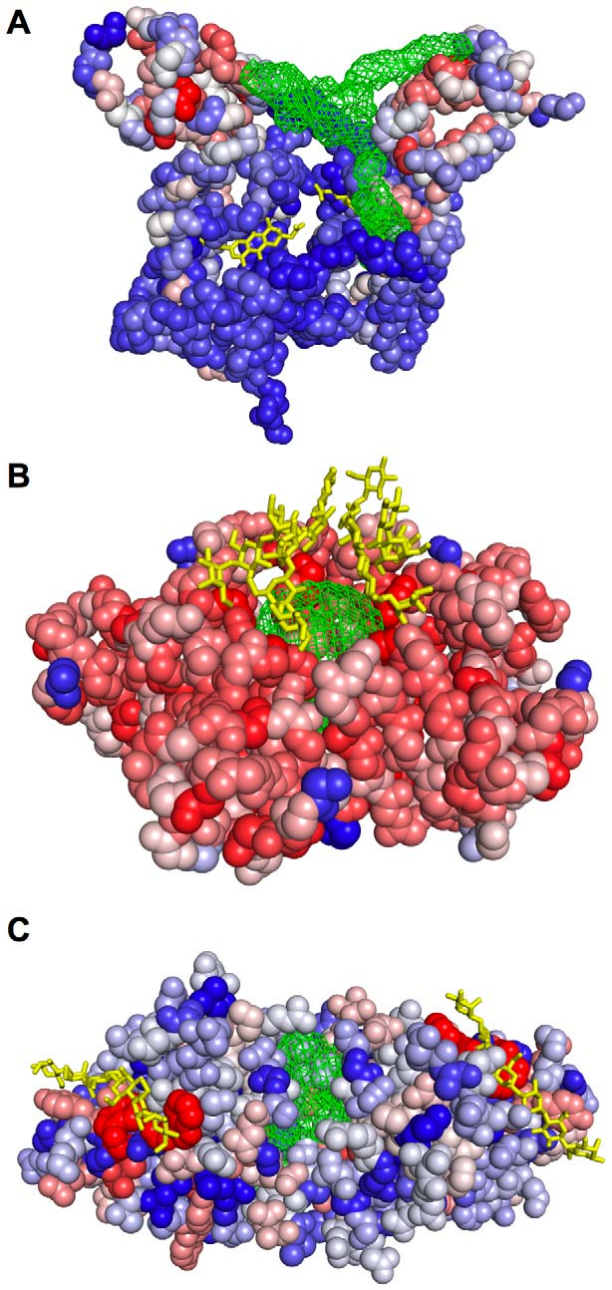
Examples of difficult structures. For each structure, evolutionary sequence conservation has been mapped to the surface of the protein backbone (all atoms in pane (C)) with warmer colors indicating greater conservation. Bound ligands are shown in yellow, and the pocket predictions of *ConCavity* are represented by green meshes. (A) The ActR protein (PDB: 3B6A) contains both a ligand-binding (bottom half) and a more conserved DNA-binding domain (top half). (B) The ring-shaped pentameric B-subunit of a shiga-like toxin (PDB: 1CQF) binds globotriaosylceramide (Gb3) via a relatively flat interface that surrounds the center of the ring. (C) The carbohydrate binding sites of the CBM29 protein (PDB: 1GWL) are too long and flat to be detected by *ConCavity* in the presence of a concave pocket between the chains. As illustrated here, *ConCavity*'s inaccurate predictions are often the result of misleading evolutionary sequence conservation information (A) or ligands that bind partially or entirely outside of well-defined concave surface pockets (B, C). In (A) and (B), *ConCavity* misses the ligands, but identifies functionally relevant binding sites for other types of interactions (DNA and protein).

While ConCavity signficantly outperforms previous approaches, its performance is not flawless. In Figure 7, we give three example structures that illustrate patterns observed when *ConCavity* performs poorly. Handling these cases is likely to be important for further improvements in ligand binding site prediction.

The first pattern common among these difficult cases is evolutionary sequence conservation information leading predictions away from actual ligand binding sites. Figure 7A provides an example in which the ligand binding site is less conserved than other parts of the protein. The ActR protein from *Streptomyces coelicolor* (PDB: 3B6A) contains both a small molecule ligand-binding and a DNA-binding domain [63]. The ligand-binding domain is in the bottom, less-conserved half of the structure. The DNA-binding domain is found in the more conserved top half of the given structure. The greater conservation of this domain causes *ConCavity* to focus on the DNA-binding site over the ligand binding site. In other cases, conservation information is uninformative due to a lack of homologous sequences. Conservation estimates based on low quality sequence alignments may harm performance for some structures, but we have found that they still provide a net performance gain overall (Text S1).

Figure 7 also provides two examples of another difficult case: ligands bound outside of clearly defined, concave surface pockets. In Figure 7B, *ConCavity* identifies the center of the ring-shaped structure of the pentameric B-subunit of a shiga-like toxin (PDB: 1CQF) as the binding site. This protein binds to glycolipids, like the globotriaosylceramide (Gb3) shown, via a relatively flat interface that surrounds the center of the ring [64]. The center cavity (*ConCavity*'s prediction) is filled by a portion of the A-subunit of the toxin (not included in the structure) which after binding breaks off and enters the host cell. Figure 7C shows the structure of a dimeric noncatalytic carbohydrate binding module (CBM29) from *Piromyces equi* complexed with mannohexaose (PDB: 1GWL). The carbohydrate ligands bind in long flat clefts on the protein surface [65]. Even though these sites exhibit significant evolutionary conservation, their geometry prevents them from being predicted. Instead, a less conserved pocket formed between the chains is highlighted by *ConCavity*.

Overall, cases such as these are rare; *ConCavity*'s predictions fail to overlap a ligand in only 5% of structures. In addition, some of these “incorrect” predictions are actually functionally relevant binding sites for other types of interactions as illustrated in Figure 7.

### Integrating conservation and structure improves prediction of catalytic sites

Ligand-binding sites are not the only type of functional site of interest to biologists. A large amount of attention has been given to the problem of identifying catalytic sites. As noted above, the majority of enzyme active sites are found in large clefts on the protein surface, so even though the structural methods considered in this paper were not intended to identify catalytic sites, they could perform well at this task.

Table 5 gives the results of an evaluation of the methods' ability to predict catalytic sites (defined by the Catalytic Site Atlas [66]) in the LigASite apo dataset. Compared to ligand binding site prediction, the relative performance of the methods is different in this context. The *ConCavity* approach still significantly outperforms the others (p<2.2e−16 for *Structure*, p = 8.223e−4 for *Conservation*). Most surprisingly, *Conservation* significantly outperforms methods based on structure alone (p = 9.863e−3 *Ligsite^+^*, p = 4.694e−6 *Pocketfinder^+^*, p = 1.171e−6 *Surfnet^+^*). All the methods have lower PR-AUC when predicting catalytic sites than predicting ligand-binding residues (e.g., *ConCavity^L^* has PR-AUC of 0.315 versus 0.608); this is due in large part to the considerably smaller number of catalytic residues than ligand-binding residues per protein sequences.

**Table 5:** Catalytic residue identification (LigASite apo).

Method	PR-AUC
*ConCavity^L^*	0.315
*ConCavity^P^*	0.301
*ConCavity^S^*	0.288
*Conservation*	0.249
*Ligsite^+^*	0.190
*PocketFinder^+^*	0.149
*Surfnet^+^*	0.142
*Random*	0.012

*ConCavity* identifies more catalytic sites than other methods. However, in contrast to ligand binding residue prediction, *Conservation* outperforms the structure-based approaches at detecting catalytic sites.

These results imply that being very evolutionarily conserved is more indicative of a role in catalysis than being found in a surface pocket. Though catalytic sites are usually found in pockets near bound ligands, there are many fewer catalytic sites per protein than ligand-binding residues. As a result simply searching for residues in pockets identifies many non-catalytic residues. This is consistent with earlier machine learning studies that found conservation to be a dominant predictive feature [5],[49],[50], and it suggests that new structural patterns should be sought to improve the identification of catalytic sites.

Several previous methods have combined sequence conservation and structural properties in machine learning frameworks to predict catalytic sites [5],[50],[51]. Direct comparison with these methods is difficult because most datasets and algorithms are not readily available. Tong et al. [51] compared the precision and recall of several machine learning methods on different datasets in an attempt to develop a qualitative understanding of their relative performance. While it is not prudent to draw conclusions based on cross-dataset comparisons, we note for completeness that *ConCavity*'s catalytic site predictions the diverse LigASite dataset achieve higher precision (23.8%) at full recall than the maximum precision (over all recall levels) reported for methods in their comparisons.

## Discussion

Evolutionary sequence conservation and protein 3D structures have commonly been used to identify functionally important sites; here, we integrate these two approaches in *ConCavity*, a new algorithm for ligand binding site prediction. By evaluating a range of conservation and structure-based prediction strategies on a large, diverse dataset of ligand binding sites, we establish that structural approaches generally outperform sequence conservation, and that by combining the two, *ConCavity* outperforms conservation-alone and structure-alone on about 95% and 70% of structures respectively. Overall, *ConCavity*'s first predicted residue contacts a ligand in nearly 80% of the apo structures examined, and it maintains high precision across all recall levels. These results hold for the three variants of *ConCavity* we considered, each of which uses a different underlying structure-based component. In addition, *ConCavity*'s integrated approach provides significant improvement over conservation and structure-based approaches on the common task of identifying drug binding sites.

Combining sequence conservation-based methods with structure information is especially powerful in the case of multi-meric proteins. Our analysis has shown that the performance of structural approaches for identifying ligand binding sites dramatically decreases as the number of chains in the structure increases; conservation alone outperforms structure-based approaches on proteins with five or more chains. It is difficult to determine from structural attributes alone if a pocket formed at a chain interface binds a ligand or not. However, ligand binding pockets usually exhibit high evolutionary sequence conservation. *ConCavity*, which takes advantage of this complementary information, performs very well on multi-chain proteins; the presence of many non-ligand binding pockets between chains has little effect on its performance.

While *ConCavity* outperforms previous approaches, we have found two main causes of poor results: misleading evolutionary sequence conservation information and ligands that bind partially or entirely outside of well-defined concave surface pockets. Ligand binding sites may lack strong conservation for a number of reasons: the underlying sequence alignment may be of low quality, there may be other more conserved functional regions in the protein, and some sites are hypervariable for functional reasons [67]. The alignment quality issue will become less relevant as sequence data coverage and conservation estimation methods improve. The second two cases may require the integration of additional features to better distinguish different types of functional sites. Similarly, finding biologically relevant ligands that bind outside of concave surface pockets will likely require the development of additional structural descriptors. Missing or incomplete ligands also affect the apparent performance of the methods, but such issues are unavoidable due to the nature of the structural data.

In implementing and evaluating previous 3D grid-based ligand binding site prediction approaches, we have found that the methods used both to aggregate grid values into coherent pockets as well as to map these pockets onto surface residues can have a large effect on performance. In order to focus on the improvement provided by considering evolutionary sequence conservation, the results for previous structure-based methods presented above use our new algorithms for these steps. We describe the details of our approaches in the Methods section. On a high level, the new methodologies we propose provide significant improvement by predicting a flexible number of well-formed pockets for each structure and by assigning each residue a likelihood of binding a ligand based on its local environment rather than on the rank of the entire pocket. We have used morphological properties of ligands to guide pocket creation, but the most appropriate algorithms for these steps depend strongly on the nature of the prediction task. These steps have received considerably less attention than computing grid values; our results suggest that they should be given careful consideration in the future.

We have focused on the prediction of ligand binding sites, but the direct synthesis of conservation and structure information is likely to be beneficial for predicting other types of functionally important sites. Our application of *ConCavity* to catalytic site prediction illustrates the promise and challenges of such an approach. Catalytic sites are usually found in surface pockets, but considering structural evidence alone performs quite poorly---worse than sequence conservation. Combining structure with evolutionary conservation provides a modest gain in performance over conservation alone. Protein-protein interface residues are another appealing target for prediction; much can be learned about a protein by characterizing its interactions with other proteins. However, protein-protein interaction sites provide additional challenges; they are usually large, flat, and often poorly conserved [68]. *ConCavity* is not appropriate for this task. Other types of functional sites also lack simple attributes that correlate strongly with functional importance. Analysis of these sites' geometries, physical properties, and functional roles will produce more accurate predictors, and may also lead to new insights about the general mechanisms by which proteins accomplish their molecular functions.

In summary, this article significantly advances the state-of-the-art in ligand binding site identification by improving the philosophy, methodology, and evaluation of prediction methods. It also increases our understanding of the relationship between evolutionary sequence conservation, structural attributes of proteins, and functional importance. By making our source code and predictions available online, we hope to establish a platform from which the prediction of functional sites and the integration of sequence and structure data can be investigated further.

## Methods

### *ConCavity*

This section describes the components of the *ConCavity* algorithm for predicting ligand binding residues from protein 3D structures and evolutionary sequence conservation.

**Figure 8:**
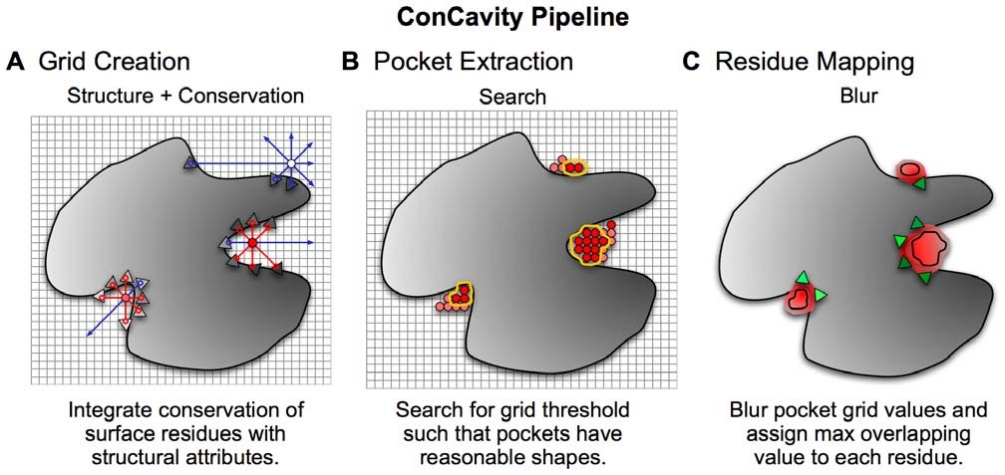
*ConCavity* prediction pipeline. The large gray shape represents a protein 3D structure; the triangles represent surface residues; and the gray gradient symbolizes the varying sequence conservation values in the protein. Darker shades of each color indicate higher values. (A) The initial grid values come from the combination of evolutionary sequence conservation information and a structural predictor, in this example *Ligsite*. The algorithm proceeds similarly for *PocketFinder* and *Surfnet*. (B) The grid generated in (A) is thresholded based on morphological criteria so that only well-formed pockets have non-zero values. For simplicity, only grid values near the pockets are shown. (C) Finally, the grid representing the pocket predictions is mapped to the surface of the protein. We perform a 3D Gaussian blur (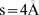) of the pockets, and assign each residue the highest overlapping grid value. Residues near regions of space with very high grid values receive the highest scores.

*ConCavity* proceeds in three conceptual steps: grid creation, pocket extraction, and residue mapping (Figure 8). First, the structural and evolutionary properties of a given protein are used to create a regular 3D grid surrounding the protein in which the score associated with each grid point represents an estimated likelihood that it overlaps a bound ligand atom (Figure 8A). Second, groups of contiguous, high-scoring grid points are clustered to extract pockets that adhere to given shape and size constraints (Figure 8B). Finally, every protein residue is scored with an estimate of how likely it is to bind to a ligand based on its proximity to extracted pockets (Figure 8C).

Grid-based strategies have been employed by several previous systems for ligand binding site prediction (e.g., [14],[16],[23]). However, our adaptations to the three steps significantly affect the quality of predictions. First, we demonstrate how to integrate evolutionary information directly into the grid creation step for three different grid-based pocket prediction algorithms. Second, we introduce a method that employs mathematical morphology operators to extract well-shaped pockets from a grid. Third, we provide a robust method for mapping grid-based ligand binding predictions to protein residues based on Gaussian blurring. The details of these three methods and an evaluation of their impacts on ligand-binding predictions are described in the following subsections.

#### Grid creation

The first step of our process is to construct a 3D regular grid covering the free-space surrounding a protein. The goal is to produce grid values that correlate with the likelihoods of finding a bound ligand at each grid point.

Several methods have been proposed to produce grids of this type. For example, *Ligsite* [16] produces a grid with values between 0 and 7 by scanning for the protein surface along the three axes and the four cubic diagonals. For each grid point outside of the protein, the number of scans that hit the protein surface in both directions---so-called protein-solvent-protein (PSP) events---is the value given to that point. A large number of PSP events indicate that the grid point is surrounded by protein in many directions and thus likely to be in a pocket.

*Surfnet* [14] assigns values to the grid by constructing spheres that fill the space between pairs of protein atoms without overlapping any other atoms. These sets of spheres are constructed for all pairs of protein surface atoms within 10 Å of each other. Spheres with a radius smaller than 1.5 Å are ignored, and spheres are allowed to have a maximum radius of 4 Å. This procedure results in a set of overlapping spheres that fill cavities and clefts in the protein. Extending the original algorithm slightly, we assign the value for each grid point to be the number of spheres that overlap it (rather than simply one for overlap and zero for no overlap as in the original algorithm). Thus, higher values are generally associated with the positions in the “center” of a pocket.

*PocketFinder* [23] assigns values to grid points by calculating the van der Waals interaction potential of an atomic probe with the protein. The Lennard-Jones function is used to estimate the interaction potential between the protein and a carbon atom placed at each grid point. The potential at a grid point 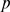 is: 
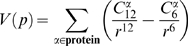
(1)where 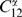 and 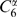 are constants (taken from AutoDock [69]) that shape the Lennard-Jones function according to the interaction energy between the carbon probe atom and protein atom 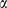, and 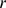 is the distance between the grid point 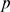 and 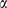 (interactions over distances greater than 10 Å are ignored).

Other grid creation methods have been proposed as well, but these three (*Ligsite*, *Surfnet*, and *PocketFinder*) provide a representative set for our study.

We augment these algorithms by integrating evolutionary information into the grid creation process. Our methodology is based on the observation that these (and other) grid creation algorithms operate by accumulating evidence (“votes”) for ligand binding at grid points according to spatial relationships to nearby protein atoms. For *PocketFinder*, each protein atom casts a “vote” for nearby grid points with magnitude equal to the (opposite of the) van der Waals potential. In *Ligsite*, every pair of protein atoms “votes” for solvent-accessible grid points on line segments between them. In our implementation of *Surfnet*, pairs of atoms “vote” for all the grid points overlapping a sphere covering the solvent accessible region between them.

Based on this observation, we weight the “votes” as the grid is created by an estimate of sequence conservation of the residue(s) associated with the atom(s) that generate the votes. We tested several schemes for scaling votes. If 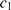 and 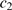 are estimated conservation scores associated with the relevant atoms (e.g., derived from their residues' conservation in multiple sequence alignments), we scaled the structure-based component by the product (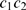), the arithmetic mean (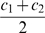), the geometric mean (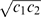), the product of exponentials (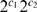), and the product of exponentials of transformed conservation values (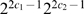). Each of these schemes provides improvement for all methods, but due to method specific differences, no single weighting scheme is best for all methods. Specifically, for *PocketFinder*, which has only one atom associated with each vote, we scale the vote (van der Waal's potential) of each atom linearly by 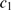. For *Ligsite* we scale the votes by arithmetic mean of the conservation values and for *Surfnet* by the product of the exponentials of the transformed conservation values.

In our study, conservation scores are calculated by the Jensen-Shannon divergence (JSD) with sequence weighting and a gap penalty [2]; however, any sequence conservation measure that produces residue scores (which are then mapped to atoms within the residues) could be incorporated.

*Performance*. The superior performance of our *ConCavity* grid creation method at predicting ligand binding pockets and residues is demonstrated in Figure 5 of the Results section. The only difference between the *ConCavity* methods (*ConCavity^L^*, *ConCavity^S^*, *ConCavity^P^*) and their counterparts based on structure alone (*Ligsite^+^*, *Surfnet^+^*, *PocketFinder^+^)* is the use of sequence conservation in the grid creation step. For each grid creation strategy, considering evolutionary conservation yields significant improvement.

#### Pocket extraction

The second step of our process is to cluster groups of contiguous, high-scoring grid points into *pockets* that most likely contain bound ligands.

Several methods have been previously proposed to address this problem. The simplest is to apply a fixed threshold to the grid, i.e., eliminate all grid points below some given value. Then, the remaining grid points can be clustered into pockets (e.g., connected components), and small pockets can be discarded. This method, which we call “*Threshold*”, has been used in previous versions of *Ligsite* [1],[16]. A problem with this approach is that the threshold is set to the same value for all proteins, which provides no control over the total number and size of pockets predicted by the algorithm. In the worst case, when every grid value is below the threshold, then the algorithm will predict no pockets. On the other hand, if the threshold is too low, then there will be many large pockets. Different proteins have different types of pockets, so no one threshold can extract appropriately sized and shaped pockets for all of them.

A slightly more adaptive method is used in *PocketFinder* [23]. In “*StdDev*” the mean and standard deviation of values in the grid are used to determine a different threshold for every protein. Specifically, the grid is blurred with 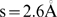, and then the threshold is set to be 4.6 standard deviations above the mean of the grid values. This approach is problematic because the threshold depends on the parameters of the grid; any change to how the protein is embedded in the grid (e.g., orienting the protein differently, changing the distance between the protein and the grid boundary, etc.) will affect the mean and standard deviation of the grid values, which in turn will affect the threshold chosen to extract pockets. For example, simply making the extent of the grid 10% greater will include a large number of near-zero values in the grid, which will bring the threshold down and make the extracted pockets larger. Also, no control is provided over the number and size of pockets; it is possible that for some proteins no grid values are 4.6 standard deviations above the mean, in which case no pockets will be predicted.

It is difficult to control the number, sizes, and shapes of extracted pockets with *Threshold* and *StdDev*. In both methods a threshold is applied to every grid point independently and clusters are formed only on the basis of geometric proximity between grid points, so it is possible to extract a set of pockets that have biologically implausible shapes. For example, there is no way to guarantee that the algorithm won't extract one very large pocket that covers a significant fraction of the protein surface, or many small pockets distributed across the protein surface, and/or pockets that contain long, thin regions where the cross-sectional diameter is too small to fit a bound ligand. Of course, it is possible to trim/discard such pockets after they have been extracted according to geometric criteria using post-processing algorithms [1],[23],[56]. However, unless there is feedback between the method used to select a grid threshold and the method used to cull pockets, then there is no way to guarantee that a biologicaly plausible set of pockets is output, i.e., it is possible that none of the pockets extracted with the chosen grid threshold meet the culling criteria.

In *ConCavity*, we integrate extraction and culling of pockets into a single framework. We perform a binary search for the grid threshold that produces a culled set of pockets that have specified properties (maximum number of pockets, total volume of all pockets, minimum volume for any pocket, minimum cross-sectional radius for any pocket, and maximum distance from protein surface). Specifically, for each step of the binary search, we select a grid threshold, extract a set of pockets (connected components of grid points having values above the threshold), and then apply a sequence of culling algorithms to trim/discard pockets based their sizes and shapes. The algorithm iterates, adjusting the threshold up or down, if the set of pockets resulting from the culling operations does not meet the specified global properties. The binary search terminates when it has found a set of pockets meeting all of the specified properties or determines that none is possible. We call this method “*Search*”.

Specifically, the culling steps are implemented with a series of grid-based filters, each of which runs in compute time that grows linearly with the size of the grid. Given a current guess for the grid threshold, the first filter simply zeroes all grid points whose value is below the threshold value.

The second filter zeroes grid points whose distance from the van der Waal's surface of the protein exceeds a given threshold, *max_protein_offset*. This filter is computed by first rasterizing a sphere for all atoms of the protein into a grid, setting every grid point within the van der Waal's radius of any protein atom to one and the others to zero. Then, the square of the distance from each grid point to the closest point on the van der Waal's surface is computed with three linear-time sweeps, and the resulting squared distances are used to zero grid points of the original grid if the squared distance is greater than *max_protein_offset* ^2^.

The third filter ensures that no part of a pocket has cross-sectional radius less than a given threshold, *min_pocket_radius*. This filter is implemented with an “opening” operator from mathematical morphology. Intuitively, the boundary of every pocket (non-zero values of the grid) is “eroded” by *min_pocket_radius* and then “dilated” by the same amount, causing regions with cross-sectional radius less than the threshold to be removed, while the others are unchanged. This operator is implemented with two computations of the squared distances from pocket boundaries, each of which takes linear time in the size of the grid.

The fourth filter constructs connected components of the grid and then zeros out grid points within components whose volume is less than a given threshold, *min_pocket_volume*. Connected components are computed with a series of depth-first traversals of neighboring non-zero grid points, which take linear time all together, and pockets are sorted by volume using quicksort, which takes 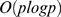 time for 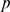 pockets.

After these filters are executed for each iteration, the total volume of all remaining pockets is computed and compared to a given target volume, *total_pocket_volume*. If the total volume is greater (less) than the target, the grid threshold is increased (decreased) to a value half-way between the current threshold and the maximum (minimum) possible threshold---initially the largest (smallest) value in the grid---and the minimum (maximum) is set to the current threshold. The process is repeated with the new threshold until the total volume of all pockets is within 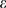 of the given *total_pocket_volume*. Note that we perform a 1 Å Gaussian blur on the *Ligsite* grid before beginning this search to provide finer control over the predicted pockets than is provided by the *Ligsite* integer grid values.

We set the parameters for these filters empirically. In previous studies, it has been observed that the vast majority of bound ligand atoms reside within 5 Å of the protein's van der Waal's surface, thus we set *max_protein_offset* to 5 Å. In order to target binding sites for biologically relevant ligands, we set *min_pocket_radius* to 1 Å and *min_pocket_volume* to 100 Å^3^. Based on the observation that the total volume of all bound ligands is roughly proportional to the total volume of the protein [17], we set *total_pocket_volume* to a given fraction of the total protein volume---2% in our studies (Text S1). Finally, we set the grid resolution to 1 Å and 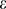 to 1 Å^3^.

*Performance*. To assess the impact of different pocket extraction strategies on the precison and accuracy of binding site detection, we implemented several alternative methods and compared how well the pockets they predict overlap with ligands in holo structures from the LigASite dataset. Table 6 shows the results of several pocket extraction algorithms (second column) on three different grids types (first column). In addition to *Thresh* and *StdDev*, *Largest(N)* refers to zeroing all grid entries not in the largest N pockets (connected components).

**Table 6:** Comparison of pocket extraction methods.

Grid Generation	Pocket Extraction	Prediction Vol. (Å^3^)	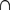 w/Lig. (Å^3^)	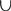 w/Lig. (Å^3^)	Prediction/Ligand	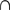 /Prediction	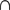 /Ligand	Jaccard coeff.
Ligsite^+^	Thresh(6)	9385.9	767.3	10595.8	4.577	0.106	0.360	0.085
Ligsite^+^	Thresh(6), Largest(3)	5919.7	674.8	7222.1	2.281	0.200	0.322	0.129
Ligsite^+^	Search	1806.8	426.9	3357.1	1.250	0.332	0.338	**0.197**
Surfnet^+^	-	44242.4	1729.3	44490.3	29.003	0.045	0.896	0.044
Surfnet^+^	Search	1766.3	426.3	3317.2	1.218	0.300	0.287	**0.166**
Pocketfinder^+^	StdDev( 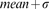 )	69477.5	1742.1	69712.6	49.250	0.028	0.900	0.028
Pocketfinder^+^	StdDev( 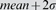 )	18317.1	1218.4	19075.9	12.026	0.094	0.652	0.085
Pocketfinder^+^	StdDev( 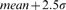 )	8303.7	896.4	9384.4	5.117	0.170	0.489	0.130
Pocketfinder^+^	StdDev( 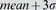 )	3703.0	591.3	5088.8	2.150	0.270	0.326	0.148
Pocketfinder^+^	Search	1807.0	436.0	3348.2	1.250	0.303	0.292	**0.167**

For three types of grids (first column), we ran different pocket extraction algorithms (second column) and compared how well the pockets overlap bound ligands in holo PQS structures. The third column (“Prediction Vol.”) lists the average volume of all predicted pockets over each protein. For reference, the average volume of all ligands observed in the PQS files (“Ligand”) is 1977.2 Å^3^. The next two columns list the average volumes of the Intersection (Ligand 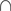 Prediction) and Union (Ligand 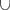 Prediction) of the Prediction and Ligand grids. Finally, the rightmost four columns list the average over-prediction factor (Prediction/Ligand), precision (Intersection/Prediction), recall (Intersection/Ligand), and Jaccard coefficient (Intersection/Union). For the last three columns, values range between zero and one, and higher values represent better performance. Comparing the average volume of the pockets predicted by each method, we see that *Search*'s pockets are closest to the actual ligand volumes. Moreover, *Search*'s high Jaccard coefficient for each grid type indicates that it provides the best tradeoff between recall and precision among the methods tested.

The statistics presented in Table 6 reflect various attributes of the pockets predicted by each extraction technique. The Jaccard coefficient (Intersection/Union) ranges between zero and one and takes into account the natural tradeoff between recall and precision by rewarding predictions that overlap the known ligands (large Intersection) and penalizing methods that predict very large pockets (large Union). Thus, it is a suitable measure for comparing the overall performance of the pocket extraction methods. For example, though the pockets of *PocketFinder^+^* with the *StdDev* (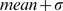) extraction method have very high recall (0.900), its Jaccard coefficient is very low, because the predicted pockets have a very large average volume (49x more than the ligands). For each grid type, our *Search* pocket extraction method predicts pockets with volumes close to the actual ligand volume and obtains the best Jaccard coefficient. As a result, we use *Search* in *ConCavity* and our implementations of previous grid based methods.

#### Residue mapping

The third step of our pipeline uses the extracted set of pockets to generate ligand-binding predictions for residues. Our goal is to score every residue based on its relationship to the extracted pockets such that residues with higher scores are more likely to bind ligands. This goal is more ambitious than that of previous residue mapping approaches which have sought only to identify the residues associated with predicted pockets.

Perhaps the simplest and most common previous method is to mark all residues within some distance threshold, 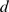, of any pocket as binding (e.g., score  = 1) and the rest as not binding (e.g., score  = 0) [25]. We call this method “*Dist-01*.” Both the pocket surface and geometric center have been taken as the reference point previously; we use the pocket surface in *Dist-01*. This approach ignores all local information about the predicted pockets. Two related methods have incorporated attributes of predicted pockets into *Dist-01*. The first assigns residues near pockets scores that reflect the size of the closest pocket (“*Dist-Size*”) [1]; residues near the largest pocket receive the highest score and so on. A similar approach uses the average conservation of all residues near the pocket (“*Dist-Cons*”) [1] to rank the pockets and assign rank-based scores to residues.

In *ConCavity*, our goal is to assign scores to residues based on their likelihood of binding a ligand. We use the original grid values (which reflect the predicted likelihood of a ligand at every point in space) to weight the scores assigned to nearby residues. Starting with the grid values within the set of extracted pockets, we blur this grid with a Gaussian filter (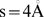), and then assign to every residue the maximum grid value evaluated at the location of any of its atoms. This method, which we call “*Blur*,” assigns residues in the same pocket different scores, since some residues are in the middle of a binding site, next to the part of a pocket with highest grid values, while others are at the fringe of a site, near marginal grid values. The score assigned by *Blur* reflects these differences in the likelihood that an individual residue is ligand binding.

In contrast to *Blur*, none of the previous residue extraction methods give different scores to residues in the same pocket. For comparison, we developed a version of the *Dist* strategy that (like *Blur)* considers the original grid values. *Dist-Raw* simply assigns to each residue within 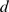 of a pocket, the value of the nearest pocket grid point.

*Performance*. We analyze the performance of these residue mapping approaches by comparing their PR-AUC on the task of predicting ligand binding residues as defined in the LigASite apo dataset. In each case we start with the same grid of extracted pockets and apply a different residue mapping algorithm. We consider all residue mapping strategies on three different pocket grids: *ConCavity^L^*, *ConCavity^S^*, and *ConCavity^P^*. For all *Dist* approaches, we set 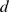 to 5 Å, and for *Dist-Cons* we consider the conservation of all residues within 8 Å of the pocket (as in [1]).

The results presented in Table 7 demonstrate that *Blur* provides better performance for each grid type than all versions of previous residue mapping approaches. Thus, we use *Blur* in *ConCavity* and our implementations of previous ligand binding site prediction algorithms. The two methods that assign residues scores based on the values of nearby grid points (*Blur* and *Dist-Raw)* provide better performance in each case than those that assign all residues in a pocket the same score based on a global property of the pocket (*Dist-Size* and *Dist-Cons)*. This suggests that the local environment around residues should be considered when predicting binding sites.

**Table 7:** Comparison of residue mapping strategies.

	Pocket Grid Source		
Mapping Method	*ConCavity^L^*	*ConCavity^P^*	*ConCavity^S^*
*Blur*	**0.608**	**0.602**	**0.587**
*Dist-Raw*	0.477	0.553	0.509
*Dist-Size*	0.442	0.486	0.474
*Dist-Cons*	0.426	0.473	0.437
*Dist-01*	0.404	0.455	0.414

We applied five residue mapping algorithms to three grids of predicted pockets (*ConCavity^L^*, *ConCavity^P^*, *ConCavity^S^* ). This table lists the PR-AUC for identifying ligand binding residues in the LigASite apo dataset for each combination. Our *Blur* algorithm achieves the best performance for each grid type.

### Previous methods

We have compared *ConCavity* to several methods for ligand binding site prediction. Many of these methods lack publicly accessible implementations, and those that are available output different representations of predicted pockets and residues. In this section, we describe of how we generate predictions for all previous methods considered in our evaluations. In some cases we have completely reimplemented strategies and in others we have post-processed the output of existing implementations. Table 8 provides a summary of these details. As mentioned earlier, a “+” appended to the method name indicates that it is (at least in part) our implementation, e.g., *Ligsite^+^*.

**Table 8:** Implementation Details of Evaluated Methods.

	Prediction Algorithm Steps			
Name	Grid Creation	Pocket Extraction	Residue Mapping	Post-processing
*ConCavity^L^*	*Ligsite+Cons*	*Search*	*Blur*	-
*ConCavity^P^*	*PocketFinder+Cons*	*Search*	*Blur*	-
*ConCavity^S^*	*Surfnet+Cons*	*Search*	*Blur*	-
*Ligsite^+^*	*Ligsite*	*Search*	*Blur*	-
*PocketFinder^+^*	*PocketFinder*	*Search*	*Blur*	-
*Surfnet^+^*	*Surfnet*	*Search*	*Blur*	-
*LigsiteCS*	http://gopubmed2.biotec.tu-dresden.de/pocket/			Residues Ranked by Pocket Rank
*Q-SiteFinder*	http://www.modelling.leeds.ac.uk/qsitefinder/			
*CASTp*	http://sts-fw.bioengr.uic.edu/castp/			
*LigsiteCSC^+^*	http://gopubmed2.biotec.tu-dresden.de/pocket/			Residues Ranked by Pocket Conservation

This table summarizes the details of each step of the ligand binding site prediction process for the methods we evaluate. The new *ConCavity* methods are based entirely on our code. We also developed new implementations (*Ligsite^+^*, *PocketFinder^+^*, and *Surfnet^+^)* of three previous methods. Predictions for the other previous methods were obtained from the listed publicly accessible web servers. These servers output sets of residues associated with predicted binding pockets. For inclusion in the residue prediction evaluation, the output of these servers was post-processed as specified. This step is not necessary for our methods, because *Blur* outputs ranked residue predictions. A “+” appended to the method name indicates that it is based (at least in part) on our code. Implementation details of each algorithm are given in the text, and code for our implementations is available online.

#### *Ligsite^+^, Surfnet^+^, and Pocketfinder^+^*

We developed new implementations of the *Ligsite*, *Surfnet*, and *Pocketfinder* methods for grid generation. This was necessary to allow us to fully integrate sequence conservation with these methods. However, it also enabled us to investigate the the effect of different pocket extraction and residue mapping algorithms on overall performance.

By default, we use *Search* to extract pockets and *Blur* to map to residues for *Ligsite^+^*, *Surfnet^+^*, and *Pocketfinder^+^*, because as was shown above, these approaches yield the best performance. Our implementations output representations of the predicted ligand binding pockets and ranked lists of contacting residues, so they can be included in both pocket and residue-based evaluations.

#### *LigsiteCS, Q-SiteFinder, and CASTp*

For our experiments, we generate binding site predictions using three publicly available web servers: *LigsiteCS* [1], *QSiteFinder* [25], and *CASTp* [19]. Each of these servers produces a list of predicted pockets represented by sets of residues. In each case, the residues do not have scores associated with them. Thus to include these methods in the ligand binding residue prediction evaluation, we must assign scores to the residues. We tried two approaches. The first assigns all predicted residues a score of one and all others a score of zero. The second ranks the residues by the highest ranking pocket to which they are assigned, i.e., all residues from the first predicted pocket are given a higher score than those from the second and so on. These approaches are similar to the residue mapping algorithms discussed in the *ConCavity* section above; however, those exact algorithms could not be applied here because the web servers do not provide representations of the full extent of predicted pockets. We found that residue ranking produces better results (data not shown), so we use this approach. We consider the default number of pockets predicted by each method: *LigsiteCS* returns three pockets; *Q-SiteFinder* returns ten pockets; and *CASTp* returns a variable number. The *Q-SiteFinder* web server would not accept proteins with more than 10,000 atoms.

*LigsiteCS*, *Q-SiteFinder*, and *CASTp* do not provide a representation of each predicted pocket's full extent, so they could not be included in the ligand location prediction evaluation.

#### *LigsiteCSC^+^*

The *LigsiteCSC* method is an extension of *LigsiteCS* that uses the evolutionary sequence conservation of residues surrounding predicted pockets to reorder the pocket predictions. This feature on the *LigsiteCS* prediction server did not work for many PQS structures in our dataset, so we implemented our own version on top of the *LigsiteCS* results. For each pocket, we calculate the average conservation of all residues within 8 Å of the pocket center. The JSD method on the HSSP alignments is used to produce the conservation scores. The top three pockets in terms of size are then ranked in terms of average conservation. This implementation follows the published description of *LigsiteCSC*, except for the use of JSD for conservation instead of ConSurf.

#### *Jensen-Shannon Divergence*

The Jensen-Shannon divergence (JSD) is used to represent the performance of evolutionary sequence conservation; it was recently shown to provide state-of-the-art performance on a range of functional site prediction tasks [2]. It compares the amino acid distribution observed in columns of a multiple sequence alignment of homologs to a background distribution. JSD scores range between zero and one. The code provided in Capra and Singh [2] with the default sequence weighting and gap penalty was used to score all alignments.

### Data

The prediction methods described in this paper take protein 3D structures and/or multiple sequence alignments as input. Protein structures were downloaded from the Protein Quaternary Structure (PQS) server [58]. Predicted quaternary structures were used (rather than the tertiary structures provided in PDB files) so as to consider pockets and protein-ligand contacts for proteins in their biologically active states. All alignments come from the Homology-derived Secondary Structure of Proteins (HSSP) database [70]. All images of 3D structures were rendered with PyMol [71].

Ligand binding sites as defined by the non-redundant version of the LigASite dataset (v7.0) [24] were used to evaluate method predictions. This set consists of 337 proteins with apo (unbound) structures, each having less than 25% sequence identity with any other protein in the set. Five of the 337 structures were left out of the evaluation: 1P5T, 1YJG, and 3DL3 lacked holo ligand information in the database, and 2PCY and 3EZM, because their corresponding holo structures are not in PQS or HSSP. Each apo structure has at least one associated holo (bound) structure in which biologically relevant ligands are identified in order to define ligand binding residues and map them to the apo structure. If multiple holo structures are available for the protein, the sets of contacting residues are combined to define the binding residues for the apo structure. We select the structures for our LigASite holo evaluation set by taking the holo structure with the most ligand contacting residues for each apo structure. The average number of holo structures for each apo structure is 2.58, and the maximum for any single structure is 32. The average chain length is 276 residues with a minimum of 59 and a maximum of 1023. The average number of positives---sites contacting a biologically relevant ligand---per chain is 25 residues (about 11% of the chain). The apo dataset includes many proteins with multiple chains; the average number of chains per protein is 2.22. The chain distribution is: 1 chain: 143, 2 chains: 112, 3 chains: 18, 4 chains: 35, 5 or more chains: 24.

The drug dataset comes from a set of 100 non-redundant 3D structures selected by [62]. This set contains a diverse set of high-quality structures (resolution <3 Å) with drug or drug-like molecules (molecular weight between 200 and 600, and 1−12 rotatable bonds) bound. Structure 1LY7 has been removed from the PDB, and 1R09 could not be parsed. We consider the 98 remaining structures.

The catalytic site annotations were taken from version 2.2.9 of the Catalytic Site Atlas [66]. There are 153 proteins in the LigASite apo dataset with entries in the Catalytic Site Atlas. These proteins have an average of 3.2 catalytic sites per chain (just over 1% of all residues in the chain).

### Evaluation

Predictions of ligand binding pockets are represented by non-zero values in a regular 3D grid around the protein. These represent regions in space thought to contain ligands. These predictions are evaluated in two ways: on the pocket level by computing their overlap with known ligands, and on the grid level by analyzing how well the grid scores rank grid points that overlap ligand atoms. We use a grid with rasterized van der Waals spheres for ligand atoms from the PQS structure as the “positive” set of grid points. From this, we calculate the intersection and union of the actual ligand atoms and the predictions. We compare methods using the over-prediction factor (Prediction Volume/Ligand Volume), precision (Intersection Volume/Prediction Volume), recall (Intersection Volume/Ligand Volume), and Jaccard coefficient (Intersection Volume/Union Volume).

We also create precision-recall (PR) curves, which compare precision (TP/(TP + FP)) on the y-axis with recall (TP/(TP + FN)) on the x-axis, to evaluate the ability of each method to predict whether a ligand atom is present at a grid point. We consider grid points that overlap a ligand atom as positives. To construct the PR curve, we calculate the precision and recall at each cutoff of the grid values in the pocket prediction grid. To summarize the performance of each method, we construct a composite PR curve [72] by averaging the precision at each recall level for each structure in the dataset. As a reference point, we include the performance of a random classifier averaged over all the structures as well. The expected performance of a random method is the number of positives over the number of all grid points. The method and code of Davis and Goadrich [73] is used to calculate the area under the PR curve (PR-AUC). The significance of the difference between methods is assessed using the Wilcoxon signed-rank test over paired performance statistics for all structures in the dataset. The significance of the difference in performance of a single method on different datasets is calculated with the Wilcoxon rank-sum test.

For the residue-based evaluation, we consider how well each method's residue scores identify ligand binding residues. Positives are those residues in contact with a ligand as defined by LigASite database. PR curves were made by calculating, for each chain, the precision and recall at each position on the ranked list of residue scores. Composite PR curves were computed as described for the grid point evaluation, but curves were first averaged over the chains in a structure and then over structures. PR curves were constructed similarly for the catalytic site analysis, but positives were defined as those residues listed in the Catalytic Site Atlas.

## Supporting Information

Text S1 Supplementary text, results, and analysis.
(0.39 MB PDF)
