# Supplementary material for: Predicting Protein Ligand Binding Sites by Combining Evolutionary Sequence Conservation and 3D Structure
Source: PLoS Comput Biol. 2009 Dec 4;5(12):e1000585. doi: 10.1371/journal.pcbi.1000585 (PMC2777313; doi:10.1371/journal.pcbi.1000585)
Supplement: Text S1 — Supplementary text, results, and analysis. (0.39 MB PDF) [file pcbi.1000585.s001.pdf]

# Predicting Protein Ligand Binding Sites by Combining Evolutionary Sequence Conservation and 3D Structure — Supplementary Material

John A. Capra Roman A. Laskowski Janet M. Thornton Mona Singh Thomas A. Funkhouser

October 28, 2009

**Abstract**

This document provides additional data and analysis in support of the main text. The first section analyzes several aspects of *ConCavity*'s performance and sensitivity to various inputs and parameters. The second section presents additional figures and data for conclusions made in the main text.

**Contact:** mona@cs.princeton.edu, funk@cs.princeton.edu

## 1 Additional Analysis

### 1.1 Sequence alignment quality's effect on ConCavity performance.

Estimates of evolutionary conservation, such as the *Jensen-Shannon divergence* (JSD), rely on multiple sequence alignments (MSAs) of homologous proteins. Alignment quality varies from protein to protein. For some proteins in the dataset, there are a large number of diverse homologous sequences, while others have only a few related sequences available.

To explore the effect of MSA quality on the performance of *ConCavity*, we partitioned the dataset based on the fraction of gaps in the associated alignments. We consider the fraction of gaps because many of the lower quality alignments contain a large number of sequence fragments that only partially cover the structure's sequence, e.g., a single domain.

For the 244 structures in the LigASite apo dataset with alignments containing fewer than 40% gaps, the area under the precision-recall curve (PR-AUC) for predicting ligand binding residues is on average 0.139 higher for the three *ConCavity* methods than their corresponding *Structure* counterparts. For the 88 alignments with more than 40% gaps, the average improvement is 0.102. The 40% threshold was selected because there was a clear drop in average performance at this level, and performance is quite similar within finer partitions of these two groups. Table 1 gives the performance of each method on these two sets.

This demonstrates that though alignment quality is important, considering evolutionary conservation can provide improvement over structure-alone, even when a high quality alignment is lacking. Fragmentary sequences provide useful information, but are not as reliable as fully aligned homologous proteins. There are cases where poor conservation estimates result in worse predictions than when considering structure alone. Since better estimates of the evolutionary constraints on residue positions lead to better ligand binding site predictions, *ConCavity* will likely become even more accurate as conservation estimation methods improve and more protein sequences become available.

**Table 1. *ConCavity*'s ligand binding residue identification (PR-AUC) improvement over *Structure* by alignment quality on LigASite apo.**

| Method | Residue PR-AUC Improvement | |
|---|---|---|
| | <40% Gaps | ≥40% Gaps |
| *ConCavity*$^L$ | 0.097 | 0.065 |
| *ConCavity*$^P$ | 0.141 | 0.095 |
| *ConCavity*$^S$ | 0.177 | 0.146 |

Each version of *ConCavity* improves over the corresponding structure-based method on both sets of alignments. The improvement is smaller for alignments of lower quality (as measured by gap percentage). *ConCavity*$^L$'s overall improvement is lower than that of the other methods, because *Ligsite*$^+$ is the best performing of the structure-based methods.

## 1.2 Sensitivity of *ConCavity*'s performance to alignment source and conservation estimation algorithm.

*ConCavity* provides a general framework for combining properties of amino acid residues, e.g., evolutionary sequence conservation, with properties of the space around the surface of a protein, e.g., being in a concave pocket. In the main text, we demonstrated that *ConCavity* performed well for a range of structure-based surface pocket identification algorithms. We now show that *ConCavity*'s performance is not tied to a single method for estimating evolutionary sequence conservation.

The alignments used to estimate evolutionary sequence conservation for all results reported in the main text come from the HSSP database. These alignments often contain a large number of sequences and sequence fragments. We demonstrated in the previous section that even very gappy alignments from HSSP provide performance improvement when used in *ConCavity*. To explore the effect of alignments with different properties on performance, we estimated sequence conservation using JSD on alignments from the ConSurfDB [1] for all proteins in the LigASite holo dataset. These alignments are generated using PSI-BLAST, MUSCLE, and a number of sequence filters. In general, they contain fewer sequences and are less gappy than their HSSP counterparts. The average PR-AUC for *ConCavity* using conservation estimates based on the HSSP alignments is 0.659 and on the alignments from ConSurf is 0.672; this difference in performance is not significant ($p$=0.234).

There are many methods other than JSD for estimating evolutionary sequence conservation. We now consider *ConCavity*'s performance when a different, state-of-the-art [2] method, Rate4Site [3], is used on the ConSurfDB alignments. *ConCavity* has a PR-AUC of 0.672 with the JSD method on the ConSurfDB alignments. When Rate4Site is used, its performance experiences only a small change (PR-AUC of 0.665).

Thus *ConCavity*'s strong performance is not specific to a particular type of multiple sequence alignment or a given method of estimating evolutionary sequence conservation.

## 1.3 *ConCavity* outperforms a direct approach to integrating information from sequence conservation and 3D structure.

Clusters of conserved residues in 3D have been found to overlap with binding sites [4, 5]. These results suggest that simply giving high scores to residues found to be near in 3D to other highly conserved residues might be sufficient to identify ligand binding sites. We implemented and evaluated a method based on this observation that combines sequence conservation with structural information. Briefly, the method, which we call *3D Neighborhood*, places the protein in a regular grid, performs a 3D Gaussian blur ($\sigma = 1.0$Å) of the conservation scores of each residue, and assigns each residue the maximum overlapping grid value after this blur. Thus residues near in space to other conserved residues receive high scores.

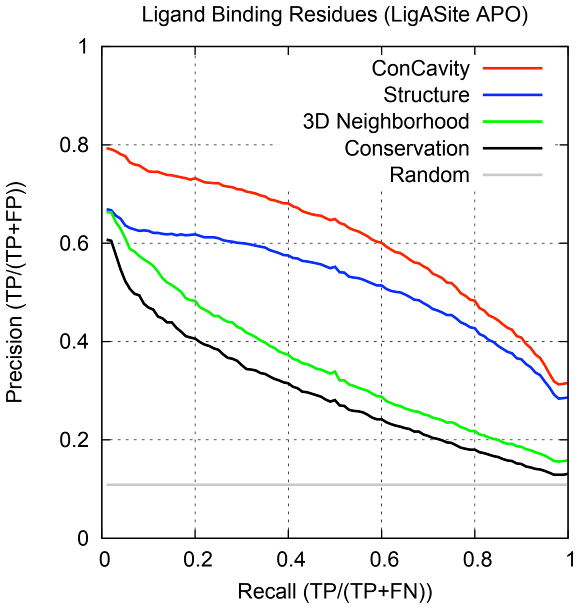


**Figure 1. Comparison of *3D Neighborhood* to other prediction strategies.** *ConCavity*'s integration of sequence and structure performs significantly better than the approach taken in *3D Neighborhood*.

Figure 1 compares the performance of *3D Neighborhood* to *ConCavity*, *Structure*, and *Conservation* on the LigASite apo dataset. *3D Neighborhood* provides some improvement over considering conservation alone, but it is not competitive with *ConCavity*. Combining sequence conservation with higher-level structural analysis, such as pocket finding, provides significant improvement over combining conservation with the raw locations of residues in space.

### 1.4 Effect of *ConCavity* Parameter Settings on Performance

The *ConCavity* method allows the user to set a number of parameters which constrain the pockets predicted. The results presented in the main text were created by setting the target predicted pocket volume to 2% of the protein volume. This section presents the motivation for this choice and explores the effect of using more accurate estimates. We also demonstrate that the ratio of protein volume to ligand volume varies among different types of proteins. We encourage users to experiment with their own application-specific settings. (Note that this analysis is based version 4.0 of the LigASite database.)

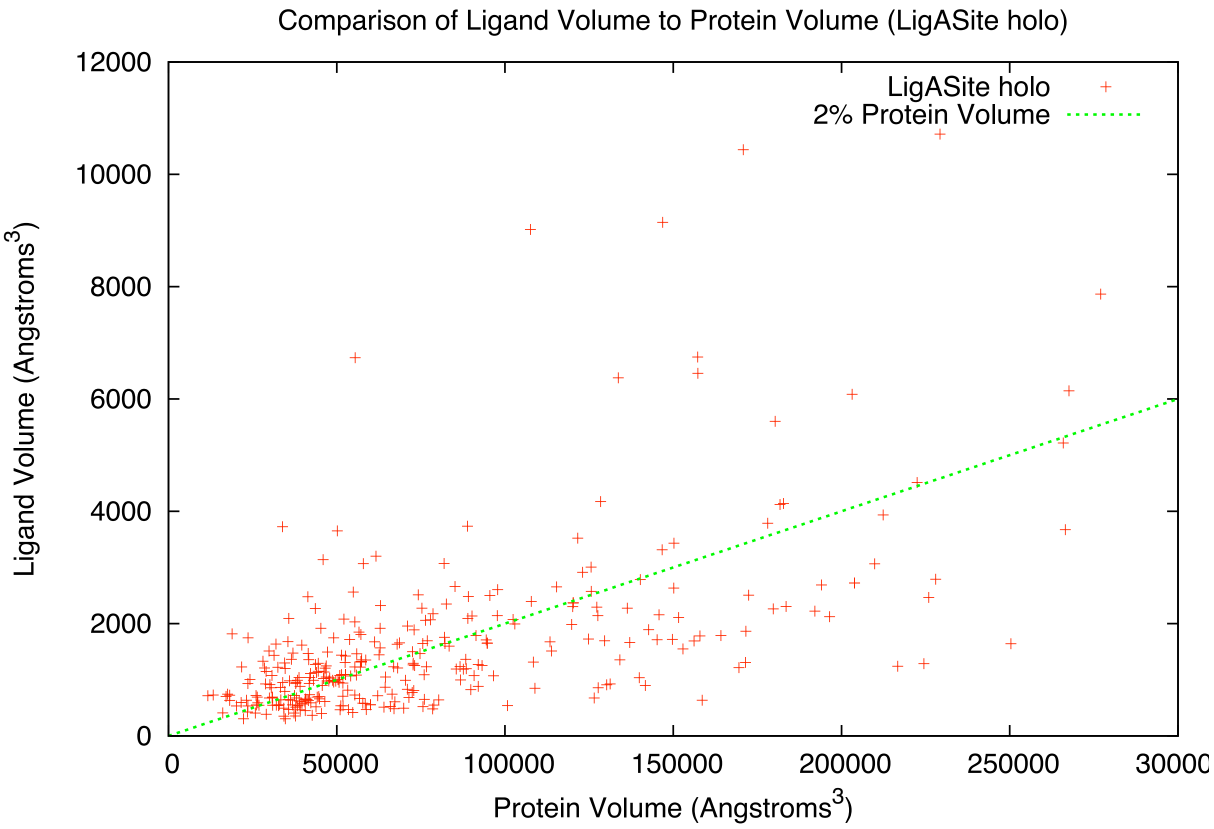


**Figure 2. Comparison of ligand volume to protein volume.** There is significant variation in the ratio of ligand volume to protein volume across the proteins of the LigASite holo dataset. Our decision to set the target prediction volume as 2% of the protein volume reflects the linear trend observed here. Table 2 shows the effect of having knowledge of the actual ligand volume prior to prediction.

**2% of the protein volume is a reasonable target volume.** Figure 2 shows a point for each structure in the LigASite holo dataset. The volume of the bound ligand(s) is plotted on the y-axis and the volume of the quaternary structure of the protein is plotted on the x-axis. A few proteins with extremely large ligands or structures are not found within the range of this plot. There is significant variation in the ratio, but there is a linear trend. The volume of the ligand(s) is unknown in real-world prediction tasks, so for simplicity, we use 2% of the protein volume as a simple heuristic for setting the target volume in *ConCavity*.

**Sensitivity of *ConCavity*'s performance to the input target prediction volume.** Table 2 investigates the sensitivity of *ConCavity* to the accuracy of the target prediction volume. $ConCavity_{Ligand}$ uses the volume of the actual bound ligand as the target volume. The average volume of the predictions of *ConCavity*

is 1786.2Å$^3$, and the average volume of the predicted pockets for $ConCavity_{Ligand}$ is 1880.8Å$^3$. In the pocket and residue PR-AUC evaluation, there is very little difference in the two methods' performance. However, in the Jaccard pocket-ligand overlap statistic, $ConCavity_{Ligand}$, with the more accurate estimate of the ligand volume, achieves better performance. However, this may simply be the result of its predictions being larger on average than *ConCavity*'s. The PR-AUC evaluations take into account the scores assigned to pocket grid points and residues, while the overlap evaluation does not. The equivalent performance of the methods in PR-AUC evaluations suggests that both give similar positions high scores, and these scores dominate the ranking of grid points and residues. Overall, this indicates that our simple heuristic is reasonable, but that better estimates of the target volume could provide modest improvement in certain prediction tasks.

**Table 2.** Effect of accuracy of target volume on *ConCavity*'s performance.

| Method | Residue PR-AUC | Grid Value PR-AUC | Jaccard coefficient |
|---|---|---|---|
| $ConCavity_{2\%}$ | 0.637 | 0.280 | 0.252 |
| $ConCavity_{Ligand}$ | 0.644 | 0.279 | 0.276 |

This table compares the average residue precision-recall AUC, the average grid value precision-recall AUC, and the average Jaccard coefficient of prediction-ligand overlap for two versions of *ConCavity*. (See the main text for more details on these statistics.) The first method uses 2% of the protein volume as the target volume for prediction, and the second uses the volume of the actual bound ligand(s). There is very little difference between the performance of the methods in the PR-AUC evaluations. The more accurate estimates provide some improvement in the ligand overlap evaluation.

**Enzymes and non-enzymes have different ratios of ligand volume to pocket volume.** We divided the proteins of the LigASite holo dataset into enzymes and non-enzymes, and calculated the volume of bound ligands to the protein volume (Figure 3). The non-enzyme proteins bind ligands that are larger (as a fraction of their volume) than the enzyme proteins. For comparison with our 2% of the protein volume heuristic, we fit lines using linear regression to the two sets of proteins. Since *ConCavity* allows users to specify target volumes for the predicted pockets, a user can use knowledge of the protein under study (e.g., enzyme/non-enzyme) to tune these parameters.

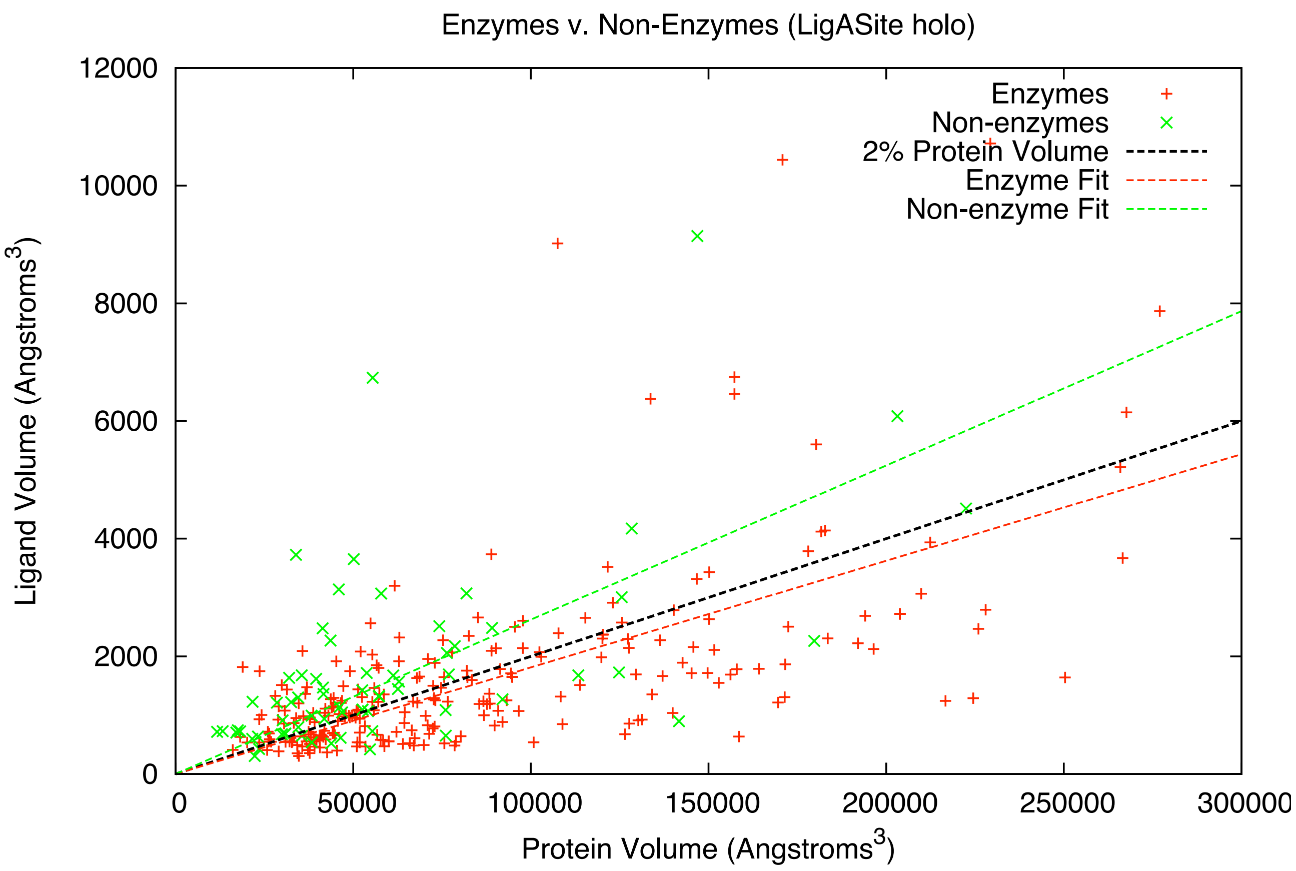


**Figure 3. Comparison of ligand volume to protein volume for enzyme and non-enzyme proteins.** In the proteins of the LigASite holo dataset, ligands found bound in non-enzyme protein structures are larger as a fraction of the overall protein volume than those bound by enzymes. For comparison with our 2% of protein volume heuristic, we fit (by linear regression) lines to the enzyme and non-enzyme data points.

# 2 Supporting Data and Results

For the sake of clarity, we did not present results for all methods in each test we performed in the main text. This section gives several tables and figures that provide additional data in support of the results given there. Table 3 lists ligand overlap statistics for all methods' predicted pockets; results for *Ligsite*$^+$ and *ConCavity*$^L$ were presented in the main text. Figures 4 and 5 show that the conclusions made in the main text about the performance of *Ligsite*$^+$ and *ConCavity*$^L$ on multi-chain proteins hold for *Pocketfinder*$^+$ and *Surfnet*$^+$ based methods as well. Specifically, methods based on structure alone perform very poorly as the number of chains in the protein structure increases, and that *ConCavity* performs well, no matter the number of chains. Figure 6 provides PR curves for the PR-AUC values reported in the comparison of performance on apo and holo structures in the main text.

**Table 3.** Comparison of the overlap between pockets predicted by each method and bound ligands in holo protein structures from the LigASite database.

| Method | Fraction with Ligand Overlap | Prediction Vol. (Å$^3$) | Ligand Vol. (Å$^3$) | Prediction ∩ Ligand (Å$^3$) | Prediction ∪ Ligand (Å$^3$) | Jaccard coefficient |
|---|---|---|---|---|---|---|
| *ConCavity*$^L$ | 0.95 | 1806.9 | 1977.2 | 647.6 | 3136.5 | 0.257 |
| *ConCavity*$^P$ | 0.95 | 1806.0 | 1977.2 | 693.8 | 3089.4 | 0.257 |
| *ConCavity*$^S$ | 0.95 | 1806.8 | 1977.2 | 686.5 | 3097.4 | 0.253 |
| *Ligsite*$^+$ | 0.92 | 1806.8 | 1977.2 | 426.9 | 3357.1 | 0.197 |
| *PocketFinder*$^+$ | 0.93 | 1807.0 | 1977.2 | 436.0 | 3348.2 | 0.167 |
| *Surfnet*$^+$ | 0.93 | 1766.3 | 1977.2 | 426.3 | 3317.2 | 0.166 |

The first column gives the fraction of proteins for which a method's predictions overlap a ligand. The second column (Prediction Vol.) lists the average volume of the predicted pockets for each protein, while the third column (Ligand Vol.) lists the average volume of ligands observed in the PQS file. The next columns give the average volumes of the Intersection and Union of the predictions and ligands and the Jaccard coefficient (Intersection / Union). The *ConCavity* and structure-only methods predict pockets of similar sizes—all use a similar pocket threshold—but the *ConCavity* methods' predictions overlap more of the bound ligands. The higher Jaccard coefficients for all *ConCavity* versions imply that they better manage the tradeoff between precision and recall.

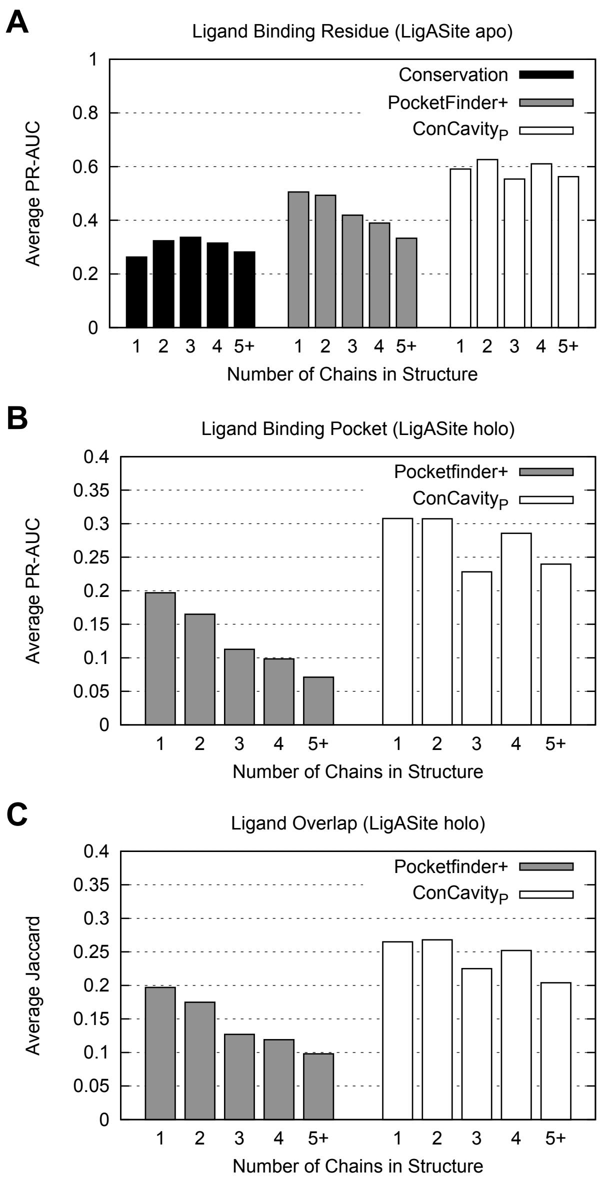


**Figure 4. Ligand-binding site identification performance by number of chains in structure for methods based on *Pocketfinder*.** **(A)** gives the average area under the precision-recall curve (PR-AUC) for predicting ligand binding residues on each set of structures; **(B)** gives the average PR-AUC for ligand binding pocket identification; and **(C)** gives the average Jaccard coefficient of the overlap of the predicted pockets with bound ligands. (See the main text for more details on these statistics.) Methods based on structure alone have an increasingly difficult time distinguishing among ligand-binding pockets and non-ligand-binding gaps between chains as the number of chains in the protein increases. This trend is clear in each type of evaluation. *Conservation*'s performance does not exhibit this effect (A). The integration of sequence conservation and pocket prediction in *ConCavity* improves performance in each chain based partition in each evaluation. *ConCavity* sees only a modest decrease in performance on proteins with multiple chains. *Conservation* alone could not be included in (B) and (C), because it does not make pocket predictions. Note that the y-axes in the figures do not all have the same scale. The number of structures per chain group: 1 chain: 143, 2 chains: 112, 3 chains: 18, 4 chains: 35, 5+ chains: 24.

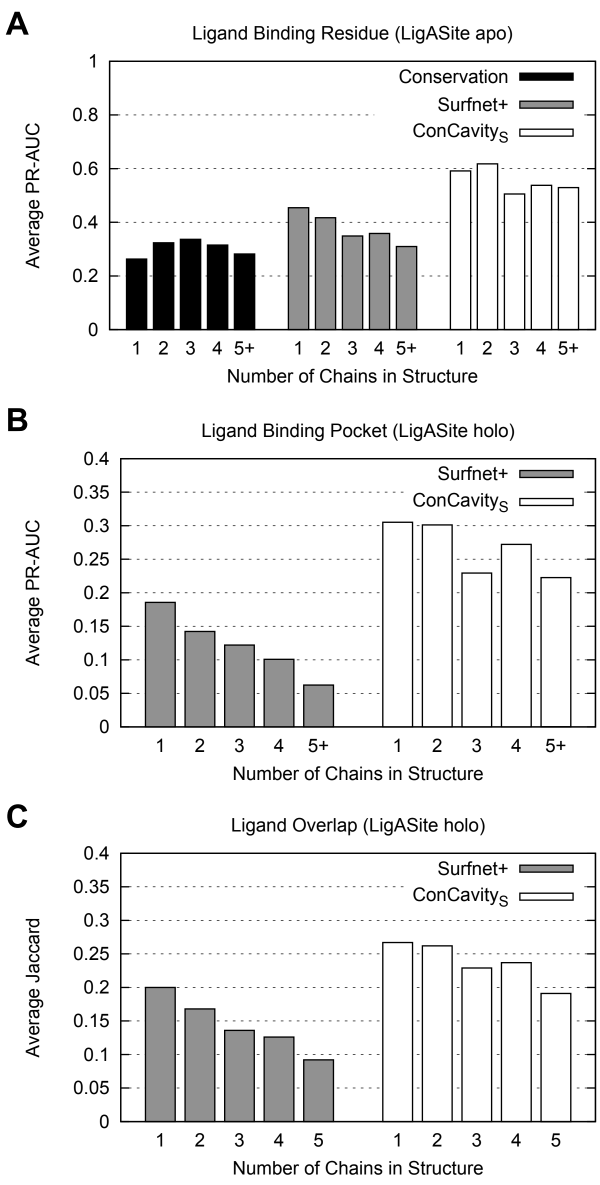


**Figure 5. Ligand-binding site identification performance by number of chains in structure for *Surfnet*-based methods. (A)** gives the average area under the precision-recall curve (PR-AUC) for predicting ligand binding residues on each set of structures; **(B)** gives the average PR-AUC for ligand binding pocket identification; and **(C)** gives the average Jaccard coefficient of the overlap of the predicted pockets with bound ligands. See Figure 4 for more discussion.

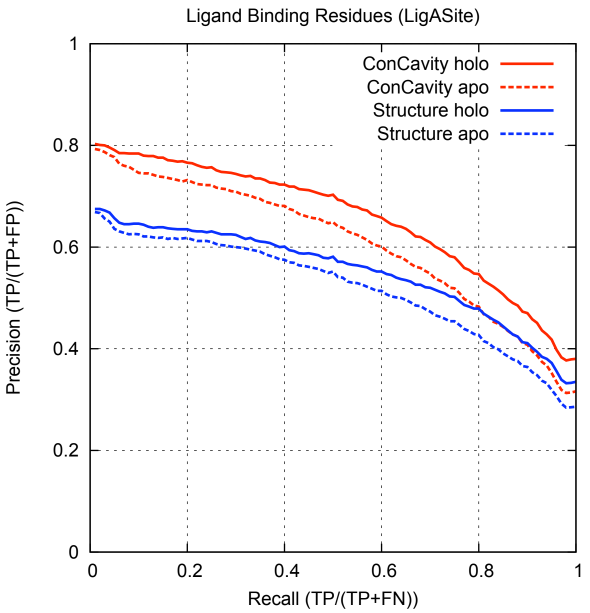


**Figure 6. Comparison of ligand-binding site identification performance on apo and holo versions of the dataset.** The performance on holo structures is given by the solid lines and the apo performance is represented by the dashed curves. Both of the methods with a structural component (*Structure* and *ConCavity*) perform worse on the apo structures. This is expected because the structural changes that occur upon binding of the ligand often define the binding site more clearly in the holo structure. However, the drop in performance is relatively small. The curves are similar for *Surfnet*$^{+}$- and *Pocketfinder*$^{+}$-based methods.

# References

[1] Goldenberg O, Erez E, Nimrod G, Ben-Tal N (2009) The ConSurf-DB: Pre-calculated evolutionary conservation profiles of protein structures. Nucleic Acids Res 37: D323-327.

[2] Capra J, Singh M (2007) Predicting functionally important residues from sequence conservation. Bioinf 23: 1875-1882.

[3] Mayrose I, Graur D, Ben-Tal N, Pupko T (2004) Comparison of site-specific rate-inference methods: Bayesian methods are superior. Mol Biol Evol 21: 1781-1791.

[4] Yao H, Kristensen D, Mihalek I, Sowa M, Shaw C, et al. (2003) An accurate, sensitive, and scalable method to identify functional sites in protein structures. J Mol Biol 326: 255-261.

[5] Panchenko A, Konrashov F, Bryant S (2004) Prediction of functional sites by analysis of sequence and structure conservation. Prot Sci 13: 884-892.
